# FPR2 deficiency alleviates LPS-induced depressive-like behaviors in mice by suppressing the microglial CSF1/NLRP3 inflammasome pathway

**DOI:** 10.1186/s12974-026-03963-4

**Published:** 2026-07-16

**Authors:** Hanqi Wang, Yijun Chen, Junna Chang, Zhen Liu, Yang Yu

**Affiliations:** Shanghai Frontiers Science Center of Drug Target Identification and Delivery, Engineering Research Center of Cell and Therapeutic Antibody, Ministry of Education, School of Pharmaceutical Sciences, Shanghai Jiao Tong University, Shanghai, 200240 China

**Keywords:** Depression, Anxiety, Microglia, Formyl peptide receptor 2 (FPR2), NOD-like receptor protein 3 (NLRP3), Colony-stimulating factor 1 (CSF1), Serum amyloid A (SAA), Neuroinflammation, Lipopolysaccharide (LPS), RNA sequencing

## Abstract

Depression is one of the most prevalent psychiatric disorders worldwide, yet its pathogenesis remains unclear. Here, we aimed to investigate the effects of formyl peptide receptor 2 (FPR2), a key regulator of innate immunity and inflammation, on lipopolysaccharide (LPS)-induced depression-related behaviors in mice after intraperitoneal administration, and to elucidate its regulatory mechanisms in microglia. FPR2 knockout (*Fpr2*^–/–^) significantly attenuated LPS-induced depressive and anxiety-like behaviors in mice. LPS markedly increased FPR2 expression in microglia of the prefrontal cortex (PFC) and hippocampus, while only a minimal increase was observed in neurons. FPR2 deficiency alleviated LPS-induced microglial activation and reduced neuronal synaptic alterations. RNA sequencing and validation experiments confirmed that FPR2 deletion substantially decreased LPS-induced microglial NLRP3 inflammasome activation and IL-1β levels in the brain. Mechanistically, FPR2 regulated downstream NLRP3 activation by modulating CSF1, and FPR2/CSF1 activation was governed by its upstream ligand, serum amyloid A (SAA). Analysis of public clinical datasets revealed that SAA1 levels were significantly upregulated in the orbital ventral PFC of patients with major depressive disorder (MDD) and in the plasma of patients with late-life depression. These findings demonstrate that the SAA/FPR2/CSF1/NLRP3 pathway mediates LPS-induced depressive-like behaviors by regulating microglial activation and neuroinflammation.

**Graphical abstract:**

**Figure:**
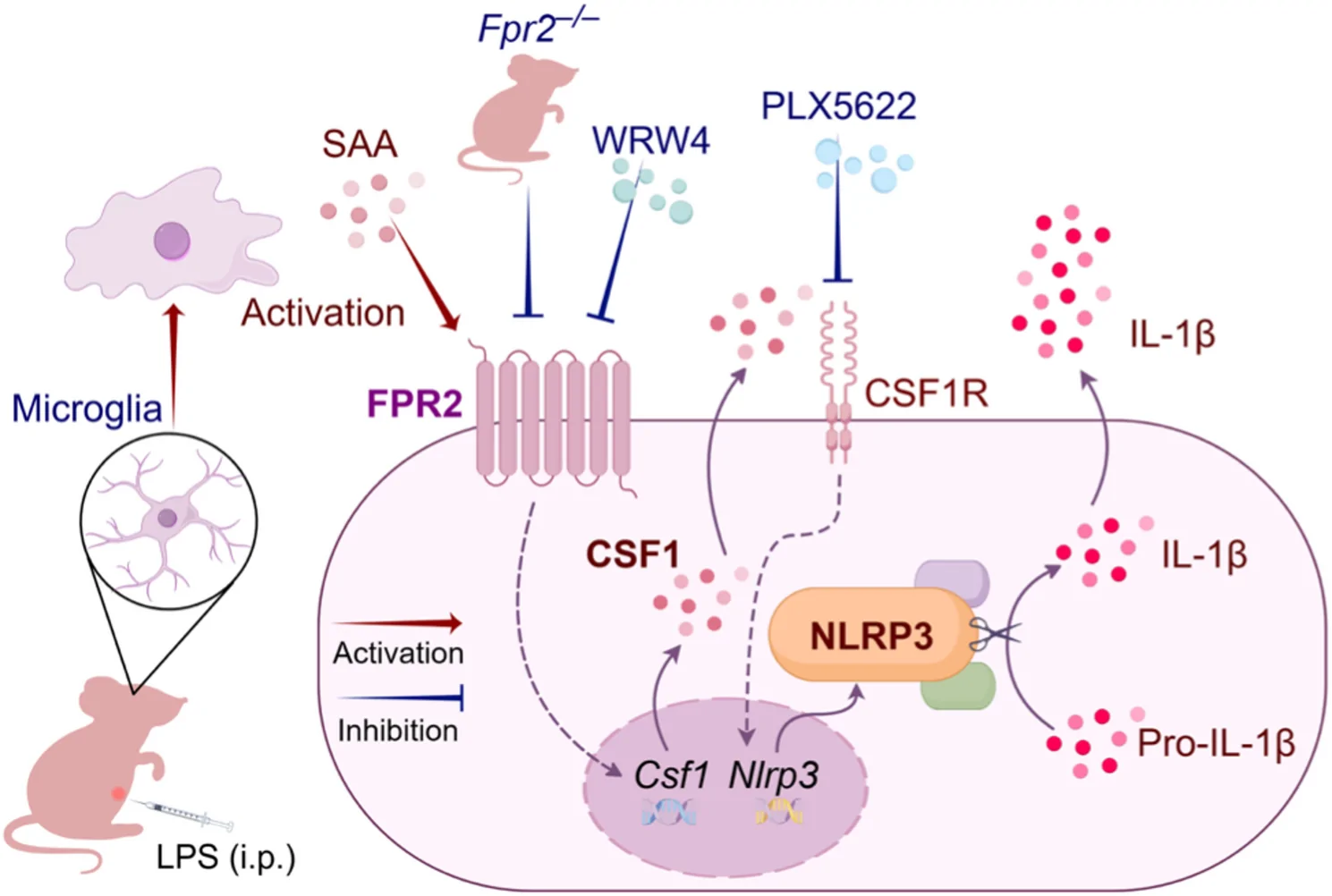

**Supplementary Information:**

The online version contains supplementary material available at https://doi.org/10.1186/s12974-026-03963-4.

## Background

Depression, a highly common psychiatric disorder worldwide [1], has an incompletely elucidated pathogenesis. The traditional monoamine neurotransmitter hypothesis fails to account for the complexity and heterogeneity of the disease [2]. Microglia, the resident immune cells of the central nervous system (CNS), have been identified as key participants in the pathophysiology of depression [3]. Clinical studies have reported that positron emission tomography imaging shows elevated levels of microglial activation markers (e.g., TSPO) in brain regions such as the prefrontal cortex (PFC) and anterior cingulate cortex (ACC) of patients with major depressive disorder (MDD), and these levels are closely correlated with the severity of depression [4, 5]. Post-mortem examinations of depressed patients who died by suicide have revealed increased microglial density and enhanced release of pro-inflammatory cytokines in the brain [6, 7]. Further evidence from preclinical animal models of depression confirms that both acute stress (e.g., lipopolysaccharide (LPS) administration) and chronic stress (e.g., social defeat and unpredictable stress) can induce microglial polarization toward a pro-inflammatory phenotype, which contributes to the development of depressive-like behaviors by releasing pro-inflammatory cytokines such as IL-1β and TNF-α [3, 8].

The NLRP3 inflammasome is a crucial pathway that promotes microglia-related neuroinflammation and contributes to the development of depression [9]. It is recognized as a key regulator of pro-inflammatory cytokine maturation and release and is mainly expressed in microglia within the brain [10, 11]. Stimuli such as stress or infection can induce the assembly of microglial NLRP3 with ASC and procaspase-1 into a complex; activated caspase-1 then cleaves pro-IL-1β into its mature form, leading to substantial release. Excessive IL-1β inhibits hippocampal neurogenesis and impairs synaptic plasticity, thereby triggering depression-related behavioral and cognitive impairments [12, 13]. Microglia, particularly the NLRP3 inflammasome pathway, are promising therapeutic targets for major depression. As the resident immune cells of the CNS, microglia recognize and transduce upstream signals through surface receptors, serving as a key “switch” to initiate microglial activation and downstream signaling, such as the NLRP3 inflammasome pathway. Receptors such as toll-like receptor 4 (TLR4) and CX3C motif chemokine receptor 1 (CX3CR1) facilitate microglial activation and contribute to the onset or exacerbation of depression by recognizing pathogen-associated molecular patterns (PAMPs), damage-associated molecular patterns (DAMPs), or neuroimmune signals [14, 15]. However, the high heterogeneity of depression suggests the existence of uncharacterized upstream regulatory pathways.

Recently, formyl peptide receptor 2 (FPR2), a G protein-coupled receptor (GPCR) with unique functions, has gained increasing attention for its roles in central immune regulation and psychiatric disorders [16]. FPR2 belongs to the formyl peptide receptor family, with FPR1 and FPR3 as core members, and exhibits distinct cell- and tissue-specific expression patterns [17]. In the CNS, FPR2 is expressed in microglia (including capillary-associated macrophages (CAMs)) and neurons [18, 19]. Peripherally, it is broadly distributed among immune cells, such as monocytes and neutrophils [20]. As a regulator of innate immunity and inflammatory responses, FPR2 initiates downstream signaling pathways upon recognition of diverse ligands, thereby modulating biological processes such as cell chemotaxis, migration, and the release of inflammatory mediators [21–23]. Its most notable feature is the diversity and complexity of its ligands: endogenous ligands include annexin A1 (ANXA1), lipoxin A4 (LXA4), resolvin D1 (RvD1), and serum amyloid A (SAA), while exogenous ligands consist of bacterial-derived N-formyl peptides, synthetic peptides (e.g., WRW4, Boc-2, W peptide), and various molecules such as lipids and small-molecule compounds [24]. This ligand diversity provides FPR2 with functional bidirectionality, as binding to different ligands can mediate either pro-inflammatory or anti-inflammatory (pro-resolving) effects [25–29]. This functional versatility enables FPR2 to play vital roles in maintaining immune homeostasis and in pathological inflammation.

Progress has been made in understanding the role of FPR2 in depression. Our previous analysis of public transcriptomic databases from patients with MDD revealed significantly elevated FPR2 transcript levels in the orbital ventral PFC of MDD patients and in peripheral blood mononuclear cells (PBMCs) from patients experiencing acute major depressive episodes. We also confirmed that FPR2 expression is markedly increased in CAMs of the PFC and hippocampus in mice subjected to a social isolation-induced depression model. Administration of the FPR2 antagonist WRW4 (either centrally or peripherally) significantly alleviates depressive and anxiety-like behaviors, inhibits excessive microglial activation, preserves blood-brain barrier (BBB) integrity, and reduces neuronal damage and synaptic dysfunction [19]. Other studies have shown that FPR2/3 knockout mice exhibit resistance to corticosterone-induced depressive-like behaviors and cognitive deficits [30]. Additionally, intracerebroventricular (ICV) injection of RvD1, an anti-inflammatory ligand of FPR2, exerts antidepressant effects in both LPS-induced and chronic unpredictable stress models [31, 32]. These findings suggest that FPR2 dysfunction is closely related to the pathogenesis of depression, yet the upstream and downstream molecular mechanisms through which FPR2 participates in depression are not fully understood. In particular, how FPR2, as a ligand-dependent receptor, regulates microglial activation and downstream inflammatory pathways (e.g., the NLRP3 inflammasome), as well as the specific roles of endogenous ligands, remains to be clarified. Therefore, this study aims to clarify the function of FPR2 in the LPS-induced acute depression model and to uncover the molecular mechanisms by which FPR2 regulates microglial activation and the NLRP3 inflammasome pathway.

In this study, we first generated FPR2 knockout mice and established an LPS-induced depression model to validate the impact of FPR2 deficiency on depressive and anxiety-like behaviors. We then measured changes in FPR2 expression in mouse microglia after LPS challenge. We also isolated microglia for RNA sequencing (RNA-seq) to elucidate the effects of FPR2 knockout on the microglial transcriptome. Additionally, we explored the upstream and downstream regulatory mechanisms of FPR2 through in vivo and in vitro experiments and found that SAA, an upstream ligand, activates the CSF1/NLRP3 pathway via FPR2. Finally, we analyzed SAA expression patterns in different brain regions and in plasma from patients with MDD using public transcriptomic databases, providing evidence for the clinical relevance of this pathway.

## Methods

### Antibodies and reagents

Detailed information on all primary antibodies used in this study is provided in Table 1. The fluorescently conjugated and HRP-conjugated secondary antibodies for immunofluorescence and Western blot are listed in Table 2. The FPR2 antagonist WRW4 (> 95% purity) was synthesized by QYAOBIO (Shanghai, China). The CSF1R antagonist PLX5622 (> 98% purity, Cat. No. P414234) was obtained from Shanghai Aladdin Biochemical Technology Co., Ltd. (Shanghai, China). Recombinant human Apo-SAA protein (rh-SAA; Cat. No. 300 − 13) was purchased from PeproTech Inc. (Rocky Hill, NJ, USA). According to the manufacturer, this protein is a consensus SAA sequence based on human Apo-SAA1α, with an N-terminal methionine and two amino acid substitutions: asparagine for aspartic acid at position 60 and arginine for histidine at position 71 (both substituted residues are present in the SAA2β isoform). Thus, it combines sequence features of both SAA1 and SAA2 isoforms. For simplicity, we refer to it as rh-SAA. The BCA protein assay kit, RIPA lysis buffer, and 4,6-diamidino-2-phenylindole (DAPI) were from Beyotime Biotech Inc. (Nantong, China). The 5× SDS-PAGE loading buffer and Tri-color pre-stained protein marker (10–180 kDa) were from Shanghai Epizyme Biomedical Technology Co., Ltd. (Shanghai, China). The High Activity Adult Brain Enzymatic Digestion Kit and CD11b+ Microglia Cell Separation Kit were obtained from RWD Life Science Co., Ltd. (Shenzhen, China). The AdeptTect Rapid PCR Kit for Mouse Tissue was from Accurate Biotechnology (Hunan) Co., Ltd. (Hunan, China). Unless otherwise specified, all other chemicals were purchased from Sigma-Aldrich, Inc. (St. Louis, MO, USA).

**Table 1:** Primary antibodies used in this study

Antibody	Source	Catalog Number
FPRL1 (FPR2)-AF647	Bioss (Beijing, China)	bs-3654R-AF647
Iba1	FUJIFILM Wako Pure Chemical (Osaka, Japan)	019-19741
Iba1	Abcam (Cambridge, UK)	ab178846
Iba1/AIF-1-AF488	Cell Signaling Technology (Danvers, MA, USA)	20,825
NeuN	Cell Signaling Technology	94,403
Synaptophysin	Proteintech (Rosemont, IL, USA)	17785-1-AP
NLRP3	Cell Signaling Technology	15,101
NALP3/CIAS1(NLRP3)-FITC	Bioss	bs-10021R-FITC
IL-1 beta	Abcam	ab254360
IL-1 beta-FITC	Bioss	bs-6319R-FITC
CSF1	Bioss	bs-4910R
SAA1 (also SAA2)	ABclonal (Woburn, MA, USA)	A14553
β-Actin	Cell Signaling Technology	4967

**Table 2:** Secondary antibodies used in this study

Antibody	Source	Catalog Number
Alexa Fluro-488-conjugated anti-rabbit IgG	Invitrogen (Waltham, MA, USA)	A21206
Alexa Fluro-568-conjugated anti-mouse IgG	Invitrogen	A10037
Alexa Fluor-647-conjugated anti-rabbit IgG	Abcam	ab150079
HRP-labeled Goat Anti-Mouse IgG	ALLWIN Biotechnology Co., Ltd. (Shanghai, China)	AW-WB1064
HRP-labeled Goat Anti-Rabbit IgG	ALLWIN Biotechnology Co., Ltd.	AW-WB1067

### Animals

*Fpr2*^−/−^ mice on a C57BL/6J background were generated by BRL Medicine Inc. (Shanghai, China). Experiments used 8-week-old male *Fpr2*^−/−^ mice and their age-matched wild-type (WT) littermates. Genotyping was performed by polymerase chain reaction (PCR) with specific primers (forward: 5’-GTC TCA ATC CGA TGC TC-3’; reverse: 5’-GGT GAA GTA GAA CTG GTG C-3′). Animals were housed in cages of 4–5 mice each under a standard 12/12 h light/dark cycle with free access to food and water. All experimental procedures were carried out in accordance with the National Institutes of Health Guide for the Care and Use of Laboratory Animals and were approved by the Biological Research Ethics Committee of Shanghai Jiao Tong University.

### LPS administration

The acute inflammatory depression model was established as previously described [33]. Briefly, 8-week-old male *Fpr2*^*−/−*^ mice and their WT littermates received a single intraperitoneal (i.p.) injection of LPS (0.83 mg/kg, dissolved in sterile saline) or an equal volume of sterile saline. Body weight was measured 24 h post-injection, after which a series of behavioral tests was conducted. Following behavioral assessments, mice were euthanized by decapitation. Brains were rapidly removed, and the prefrontal cortices (PFC) and hippocampi from the left hemispheres were dissected, snap-frozen in liquid nitrogen, and stored at − 80 °C for biochemical analyses. The right hemispheres were fixed in 4% paraformaldehyde in 0.1 M phosphate-buffered saline (PBS) for 12 h, then cryoprotected in 30% sucrose solution. Coronal sections (30 μm) were cut with a freezing microtome and stored in an antifreeze solution (12.5 g/L polyvinylpyrrolidone [average MW 40,000], 375 g/L saccharose, 375 mL/L glycol, and 625 mL/L Tris-buffered saline [TBS, 0.1 M, with 12.1 g/L Tris-base, 40 g/L NaCl]) at − 20 °C until use.

### Intracerebroventricular injection of SAA

Eight-week-old male *Fpr2*^−/−^ mice and their WT littermates were randomly assigned to receive saline, rh-SAA, or rh-SAA with PLX5622. Mice were anesthetized with sodium pentobarbital (45 mg/kg, i.p.) and positioned on a stereotaxic apparatus. A single bilateral intracerebroventricular (ICV) injection was administered, with a total of 8 µL (4 µL per side) of either 0.9% saline, 40 µM rh-SAA, or 40 µM rh-SAA with 10 µM PLX5622. The ICV injection concentration of PLX5622 (10 µM) was empirically derived from the in vitro effective concentration (1 µM) and the estimated mouse cerebrospinal fluid volume of approximately 40 µL [34]. Based on an 8-µL injection, the theoretical initial cerebrospinal fluid concentration was calculated as 5 µM. Considering unaccounted factors such as parenchymal distribution and cerebrospinal fluid turnover, we used a 2-fold higher concentration (10 µM) to ensure that the local concentration would at least transiently surpass the in vitro efficacy threshold. The coordinates relative to the bregma were: ±1.0 mm lateral, − 0.3 mm posterior, and − 1.5 mm ventral. After surgery, mice were kept on a heating pad at 37 °C until they recovered. Behavioral tests were performed three days post-surgery.

### Behavioral tests

#### Forced Swim Test (FST)

The FST was conducted in a transparent glass cylinder (18 cm diameter, 27 cm height) filled with water to a depth of 15 cm and maintained at 23–25 °C. Mice were gently introduced into the cylinder, and opaque dividers were placed between adjacent cylinders to prevent visual contact. Water was replaced after every three mice to eliminate potential interference from excreta. The total test duration was 6 min, and immobility during the final 4 min was quantified using an automated video-tracking system (TopScan, CleverSys Inc., Reston, VA, USA). Immobility was defined as the absence of active swimming, with the mouse remaining passively afloat and making only necessary movements to keep its head above water. Following the test, mice were gently dried and returned to their home cages.

#### Tail Suspension Test (TST)

Mice were suspended 35 cm above the floor with adhesive tape placed approximately 1 cm from the tip of their tails. Visual and physical contact between mice was prevented by opaque partitions. The test lasted 6 min, and immobility during the final 4 min was recorded as an indicator of behavioral despair. Immobility was defined as the absence of escape-oriented movements, including tail and limb motion. Data were acquired and analyzed using the TopScan automated video-tracking system.

#### Sucrose Preference Test (SPT)

The SPT was performed in three phases. During the adaptation phase, mice were habituated to two bottles containing 2% sucrose solution for 12 h. Subsequently, mice were deprived of both food and water for 12 h prior to the test. In the testing phase, each mouse was presented with two pre-weighed bottles, one containing 2% sucrose and the other containing tap water, for 12 h. The bottles were reweighed after the test, and sucrose preference was calculated as the percentage of sucrose solution consumed relative to total liquid intake: sucrose preference (%) = (sucrose intake / total intake) × 100%.

#### Elevated Plus Maze test (EPM)

The EPM apparatus consisted of four arms (30 × 5 cm) extending from a central platform (5 × 5 cm). Two opposing arms were open, while the other two were enclosed by 20-cm-high walls. The entire maze was elevated 80 cm above the floor. Mice were placed individually in the center, facing an open arm, and allowed to explore freely for 5 min. The number of entries into the open arms was recorded and analyzed using the TopScan automated tracking system. The apparatus was cleaned with 75% ethanol between trials to remove olfactory cues.

#### Open Field Test (OFT)

Spontaneous locomotor activity and exploratory behavior were assessed in a square polyvinyl chloride arena (50 × 50 cm, 40-cm-high walls). Each mouse was placed at the center of the arena and allowed to move freely for 5 min. Locomotion was recorded with an overhead camera connected to a computer, and total distance traveled was quantified using the TopScan system.

#### Accelerating rotarod test

Motor coordination and balance were evaluated using an accelerating rotarod. Mice were placed on a rotating cylinder, and the speed increased from 1 to 30 rpm over 90 s. The latency to fall from the rotating rod was recorded. Each mouse underwent three consecutive trials with a 5-min inter-trial interval, and the average latency was calculated for analysis.

### Cell culture and treatment

The murine N9 microglial cell line was generously provided by Dr. Yingying Le from the Institute for Nutritional Sciences at the Shanghai Institutes for Biological Sciences, Shanghai, China. Cells were cultured in IMDM supplemented with 10% fetal bovine serum (FBS), 100 U/mL penicillin, and 100 µg/mL streptomycin sulfate (all from Invitrogen) at 37 °C in a humidified atmosphere containing 5% CO_2_. For in vitro experiments, N9 cells were stimulated with LPS (1 µg/mL or 2.5 ng/mL) or rh-SAA (0.5, 1, or 1.5 µg/mL) for 24 h. In inhibitor studies, cells were pretreated with WRW4 (10 µM) for 30 min or PLX5622 (1 µM) for 15 min before stimulation, as previously reported [35, 36]. Cell lysates were collected for Western blot analysis, or cells were fixed on poly-D-lysine-coated coverslips for immunofluorescence staining.

### Immunofluorescence staining and quantitative analysis

N9 cells grown on coverslips were fixed with 4% paraformaldehyde in PBS for 20 min at room temperature. Immunofluorescence staining was then performed on fixed N9 cells or on free-floating brain sections. Briefly, samples were washed with 0.05 M TBS, permeabilized with 0.1% Triton X-100 in TBS for 15 min, and blocked with 10% normal donkey serum in TBS containing 0.5% Tween-20 for 90 min at room temperature.

Single, double, and triple labeling were performed by sequential incubation with primary and secondary antibodies, as detailed in Additional file 1: Table S1. All primary antibody incubations were carried out overnight at 4 °C, while secondary antibody and directly conjugated antibody incubations were performed for 1.5 h at room temperature. Sections were washed with TBS between each incubation step. Nuclei were counterstained with DAPI (1 µg/ml, Beyotime) for 8 min at room temperature, then washed and mounted with anti-fade mounting medium. Fluorescence images were acquired using a laser-scanning confocal microscope (Carl Zeiss, Oberkochen, Germany) with identical acquisition settings for all samples in each experiment.

To characterize microglial morphology, Z-stack images of IBA1-stained sections were captured at 3-µm intervals. Maximum-intensity projections were created and analyzed with ImageJ/Fiji (National Institutes of Health, Bethesda, MD, USA). Microglial activation was evaluated by measuring IBA1 fluorescence intensity, cell soma size, and process complexity using Sholl analysis. For Sholl analysis, the image was skeletonized, and concentric circles were placed at 5-µm intervals starting from the soma. The number of intersections at each radius was recorded using the Sholl analysis plugin in ImageJ/Fiji; a decrease in intersections indicates an activated microglial phenotype. These morphological parameters (increased soma size and reduced Sholl intersections) are well-established indicators of microglial activation [37, 38].

The expression levels of target proteins (FPR2, SYN, NLRP3, CSF1, and IL-1β) were quantified as mean fluorescence intensity within defined regions of interest (ROIs). Cell-specific expression was identified through colocalization analysis using Manders’ colocalization coefficients with the Coloc2 plugin in Fiji [19]. For instance, the M1 coefficient [M1 = Σ (S1i, coloc) / Σ (S1i)] represented the fraction of FPR2 (channel 1) fluorescence that colocalized with the microglial marker IBA1 (channel 2). Conversely, the M2 coefficient [M2 = Σ (S2i, coloc) / Σ (S2i)] indicated the fraction of IBA1 (channel 2) that colocalized with FPR2 (channel 1). Similarly, for NLRP3 and IL-1β, the M2 coefficient indicated their colocalization with IBA1, representing the proportion of these proteins within microglia.

### Western blot

Protein was extracted from mouse PFC, hippocampal tissues, or N9 cells using RIPA lysis buffer supplemented with protease and phosphatase inhibitors. Protein concentrations were determined using a BCA kit. Equal amounts of protein were heated in 5× SDS-PAGE loading buffer at 99 °C for 10 min, then separated on 10% or 12% SDS-PAGE gels and transferred to nitrocellulose membranes (GE Healthcare, Wauwatosa, WI, USA). Membranes were blocked with 5% non-fat milk for 1.5 h at room temperature and incubated overnight at 4 °C with primary antibodies against IBA1 (1:2000), NLRP3 (1:1000), CSF1 (1:1000), IL-1β (1:1000), SAA1/2 (1:1000), and β-Actin (1:2000). After incubation with HRP-conjugated secondary antibodies (Table 2), protein bands were visualized using an Amersham ImageQuant 800 imager (Cytiva, Marlborough, MA, USA) and quantified by densitometry with ImageJ software (National Institutes of Health).

### Microglia isolation

Sixteen hours after LPS or saline administration, mice were deeply anesthetized with sodium pentobarbital (40 mg/kg, i.p.) and transcardially perfused with ice-cold PBS to remove circulating blood cells. Brains were quickly harvested, and the olfactory bulbs, cerebellum, and brainstem were discarded. Brain tissue was minced into small pieces and enzymatically digested using the High Activity Adult Brain Enzymatic Digestion Kit (RWD, Cat. No. DHABE-10) following the manufacturer’s protocol. The digested tissue was gently triturated, passed through a 70 μm cell strainer (Falcon; Corning Incorporated, Corning, NY, USA), and centrifuged at 300 × g for 10 min at 4 °C. Myelin debris was removed using the High Efficiency Debris Removal Kit (RWD, Cat. No. DHDR-5006) according to the manufacturer’s instructions.

Microglia were then isolated by positive selection using the CD11b+ Microglia Cell Separation Kit (RWD, Cat. No. K1306-10). Briefly, single-cell suspensions were incubated with the anti-mouse CD11b biotin antibody for 10 min at 4 °C, washed, and then incubated with Streptavidin MicroBeads for 15 min at 4 °C. Labeled cells were applied to a magnetic column (RWD, Model HCSC-25) placed in a magnetic field. After washing to remove unlabeled cells, the column was removed from the magnetic field, and the retained CD11b+ microglia were flushed out with buffer. This protocol typically yields a highly pure (> 95%) microglial population suitable for downstream applications [39].

### RNA sequencing (RNA-seq)

RNA extraction, library construction, sequencing, and initial data processing (including clean reads filtering and FPKM quantification) were conducted by LC-Bio Technologies (Hangzhou) Co., Ltd. (Hangzhou, China). Total RNA was extracted using TRIzol reagent (Invitrogen) according to the manufacturer’s protocol. RNA quantity and purity were measured using a NanoDrop ND-1000 (NanoDrop, Wilmington, DE, USA), and RNA integrity was evaluated on a Bioanalyzer 2100 (Agilent, CA, USA), with an RIN > 7.0 as the acceptance criterion. mRNA was purified from 1 µg of total RNA using oligo(dT) magnetic beads (Dynabeads Oligo (dT)25-61005, Thermo Fisher, CA, USA) and fragmented with the Magnesium RNA Fragmentation Module (NEB, Ipswich, MA, USA). cDNA synthesis and library preparation were performed using SuperScript™ II Reverse Transcriptase (Invitrogen) and NEBNext^®^ reagents, incorporating dUTP for second-strand synthesis to enable strand-specific sequencing. Indexed libraries were size-selected with AMPureXP beads, quality-checked, and sequenced on an Illumina NovaSeq 6000 platform (LC-Bio Technology Co., Ltd., Hangzhou, China) with 2 × 150 bp paired-end reads. Raw sequencing data were preprocessed using fastp to remove adapter sequences and low-quality reads. Clean reads were aligned to the mouse reference genome (GRCm38) using HISAT2. Transcript assembly and quantification were performed using StringTie, and gene expression levels were calculated as fragments per kilobase of transcript per million mapped reads (FPKM). RNA-seq data from mouse microglia are available in the Gene Expression Omnibus (GEO) under accession number GSE334556.

### Bioinformatics analysis of RNA-seq data

Differential expression analysis between groups was performed using the R package edgeR. Genes with FPKM values below 1 in all individual samples were excluded from further analysis. Genes with an absolute fold change (|FC|) greater than 1.2 and an adjusted *p*-value less than 0.05 were considered significantly differentially expressed (DEGs). Three-dimensional principal component analysis (PCA) plots were generated using RStudio (version 4.5.1), and volcano plots were produced using GraphPad Prism 9 (San Diego, CA, USA). Gene Set Enrichment Analysis (GSEA) was conducted with the OmicStudio tool (https://www.omicstudio.cn) to identify enriched biological pathways.

To identify genes whose dysregulation was reversed by FPR2 knockout, we defined “reversed genes” as those significantly altered in the WT-LPS vs. WT-Saline comparison (|FC| > 1.2, adjusted *p* < 0.05) but showing opposite directional changes in the *Fpr2*^–/–^-LPS vs. WT-LPS comparison, with expression restored toward WT-Saline levels. Heatmaps were generated using OmicStudio tools to visualize the expression patterns of these reversed genes. These genes were then subjected to functional interaction network analysis using ClueGO (version 2.5.10) [40], a Cytoscape plugin that integrates Gene Ontology (GO) terms, KEGG, Reactome, and WikiPathways to create a functionally organized network. The reversed genes were mapped to the Reactome and WikiPathways databases. For pathway enrichment, a two-sided hypergeometric test was applied, and only pathways with *p*-value ≤ 0.05 were considered significant, with Bonferroni correction for multiple testing. The kappa score threshold was set to 0.4 to determine term-term associations, and GO tree intervals were set from min level 3 to max level 8.

Protein-protein interaction (PPI) networks for the reversed genes were constructed using the STRING database (https://string-db.org/) [41] with a confidence score > 0.4 as the cutoff. The resulting PPI network was visualized using Cytoscape (version 3.10.2). To identify hub genes within the network, topological analysis was performed using the cytoHubba [42] and CytoNCA [43] plugins. Genes with above-average scores across multiple topological metrics, including Radiality and Closeness, were selected to identify hub genes. Detailed characteristics of these hub genes are provided in Additional file 1: Tables S2 − S3.

### Real-Time quantitative PCR (RT-qPCR)

Total RNA was extracted from sorted microglia or PFC tissues using TRIzol reagent and reverse-transcribed into cDNA using a ReverTra Ace™ qPCR RT Master Mix (TOYOBO, Osaka, Japan). Quantitative PCR was conducted on a CFX96 Touch Real-Time PCR system (Bio-Rad, Hercules, CA, USA) using ChamQ Universal SYBR qPCR Master Mix (Vazyme, Shanghai, China). The PCR was performed according to the following conditions: 95 °C for 30 s, followed by 40 cycles of denaturation at 95 °C for 10 s, annealing at 60 °C for 30 s, and extension at 95 °C for 15 s, ending with a final extension at 60 °C for 60 s. Relative mRNA levels of target genes were calculated using the 2^exp(−ΔΔCt)^ method and normalized to *Actb* (*β-actin*). Primer sequences are listed in Additional file 1: Table S4.

### Analysis of brain transcriptomic and plasma proteomic datasets from individuals with depression

For brain transcriptomic data, we used 14 datasets from the NCBI Gene Expression Omnibus (GEO) database (https://www.ncbi.nlm.nih.gov/geo/), covering various brain regions from patients with major depressive disorder (MDD), as described in our publication [19]. The transcriptional expression of *SAA1/2* was analyzed using standardized, quantile-normalized values from the corresponding brain tissue datasets. GSEA was performed using the OmicStudio tool.

For proteomic data, we searched the ProteomeXchange public database (http://www.proteomexchange.org) using the keywords “Depression”, “Major depressive disorder”, or “MDD” combined with “Homo sapiens”. The inclusion criteria were: (1) studies providing raw proteomic data from human plasma or serum samples; (2) studies including both healthy controls and medication-naïve patients or those free of antidepressant or antipsychotic drugs for at least three months prior to sampling. Three datasets met these criteria and were included in the analysis: PXD034030 (late-life depression with mild anxiety symptoms) [44], PXD047302 (vascular depression) [45], and PXD050851 (postpartum depression) [46]. Detailed characteristics of these datasets are provided in Additional file 1: Table S5. Normalized protein abundance values for SAA1 were extracted and compared between depressed patients and healthy controls for each dataset.

### Quantification and statistical analysis

All data are presented as mean ± standard error of the mean (SEM). Statistical analyses were performed using GraphPad Prism 9. For comparisons among multiple groups, one-way or two-way ANOVA, followed by Tukey’s post hoc test, was used. For comparisons between two groups, an unpaired, two-tailed Student’s t-test was used. Correlations were assessed using Spearman’s correlation coefficient. A *p*-value less than 0.05 was considered statistically significant.

## Results

### FPR2 deficiency alleviates LPS-induced depressive and anxiety-like behaviors in mice

**Fig. 1:**
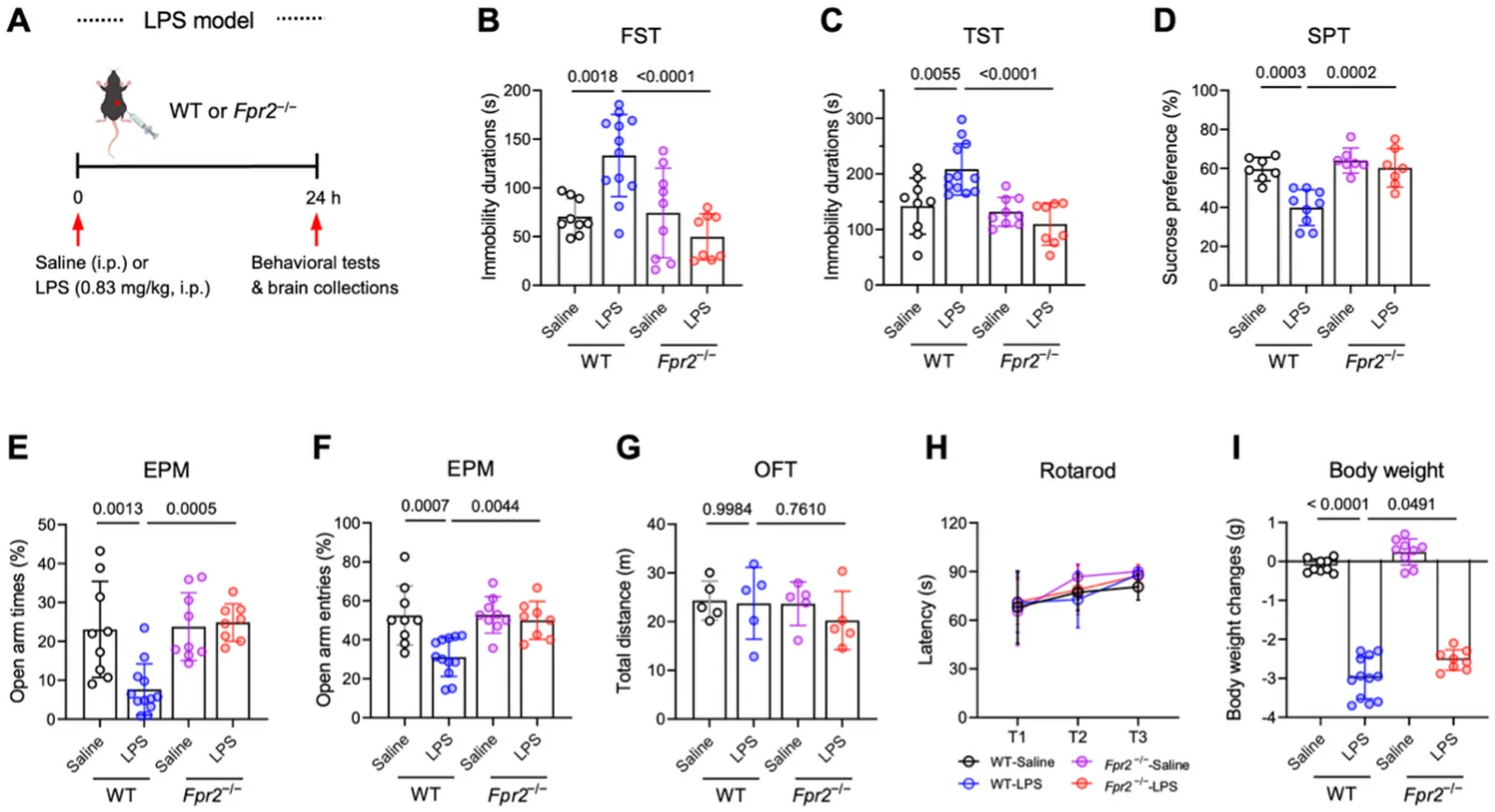
FPR2 deficiency alleviates LPS-induced depressive and anxiety behaviors in mice without affecting general locomotion or motor coordination. **A**. Schematic of the experimental timeline for establishing the LPS-induced acute depression model in wild-type (WT) and *Fpr2*^–/–^ mice. **B**,** C**. Immobility duration in the forced swim test (FST, **B**) and tail suspension test (TST, **C**) of WT and *Fpr2*^–/–^ mice 24 h after intraperitoneal (i.p.) injection of LPS or saline. **D**. Sucrose preference in the sucrose preference test (SPT) of WT and *Fpr2*^–/–^ mice 24 h after i.p. injection of LPS or saline. **E**,** F**. Percentage of time spent in open arms (**E**) and number of entries (**F**) in the elevated plus maze (EPM) of WT and *Fpr2*^–/–^ mice 24 h after i.p. injection of LPS or saline. **G**. Total distance traveled in the open field test (OFT) of WT and *Fpr2*^–/–^ mice 24 h after i.p. injection of LPS or saline. **H**. Latency to fall in the rotarod test of WT and *Fpr2*^–/–^ mice 24 h after i.p. injection of LPS or saline. **I**. Body weight changes of WT and *Fpr2*^–/–^ mice 24 h after i.p. injection of LPS or saline. Data are presented as means ± SEM, with 5–12 mice per group. One-way ANOVA followed by Tukey’s post hoc test was used for multiple comparisons. Statistical comparisons between groups are indicated by lines with *p*‑values above

Our previous study demonstrated that administration of the FPR2 antagonist WRW4 significantly ameliorates depressive and anxiety-like behaviors induced by social isolation in mice [19]. Here, we used FPR2 knockout (*Fpr2*^–/–^) mice and established an LPS-induced acute depression model (Fig. 1A) to investigate whether FPR2 is involved in acute inflammation-induced depressive-like behaviors. Eight-week-old *Fpr2*^–/–^ mice and their wild-type (WT) littermates were injected intraperitoneally with 0.83 mg/kg LPS. A series of behavioral tests was conducted 24 h post-injection. These tests included the forced swim test (FST), tail suspension test (TST), sucrose preference test (SPT), elevated plus maze (EPM), open field test (OFT), and rotarod test. In the FST and TST, immobility time was recorded to assess depressive-like behavior. The SPT was used to evaluate anhedonia by measuring sucrose preference. In the EPM, time spent in the open arms and the number of entries into the open arms were measured to assess anxiety-like behavior. The OFT assessed general locomotor activity based on total distance traveled. The rotarod test evaluated motor coordination and balance by recording the latency to fall.

We observed that, compared with the saline-injected control group, LPS administration significantly increased immobility time in the FST and TST (Fig. 1B and C), reduced sucrose preference in the SPT (Fig. 1D), and decreased time spent in and entries into the open arms in the EPM (Fig. 1E and F). These results confirm that LPS successfully induced depressive and anxiety-like behaviors in mice. Notably, FPR2 knockout reversed these LPS-induced behavioral changes (Fig. 1B–F), indicating that FPR2 deficiency alleviates LPS-induced depressive and anxiety-like behaviors. Furthermore, compared with WT littermates, FPR2 knockout did not affect immobility time in the FST and TST, sucrose preference in the SPT, or open-arm exploration in the EPM, indicating that FPR2 deletion does not influence these behaviors in the absence of LPS stimulation. Additionally, no significant differences were observed in total distance traveled in the OFT or latency to fall in the rotarod test across groups, suggesting that neither LPS administration nor FPR2 knockout affected general locomotor activity or motor coordination (Fig. 1G and H). This rules out the possibility that differences in these fundamental abilities confound the assessment of depressive and anxiety-like behaviors. Consistent with previous reports [47], LPS challenge led to a significant reduction in body weight (Fig. 1I). FPR2 knockout slightly mitigated this LPS-induced body weight loss (Fig. 1I). These findings indicate that FPR2 is involved in the pathogenesis of LPS-induced acute depression, and its deficiency can significantly ameliorate the associated depressive and anxiety-like behaviors.

### FPR2 expression is significantly upregulated in microglia following LPS administration

**Fig. 2:**
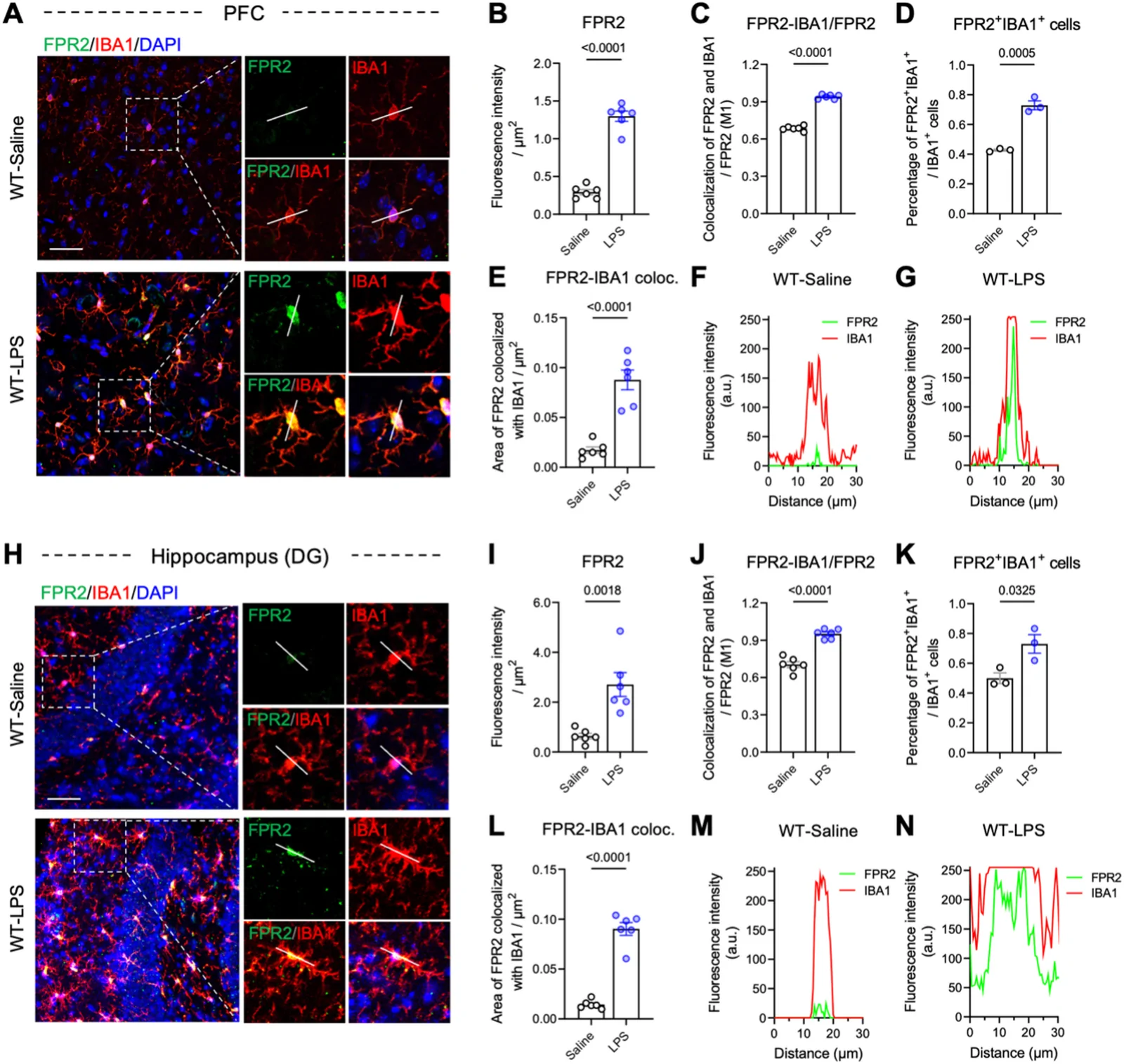
FPR2 expression is significantly upregulated in microglia of the PFC and hippocampal DG after LPS administration. **A**,** H**. Representative immunofluorescence images showing FPR2 expression in the prefrontal cortex (PFC, **A**) and hippocampal dentate gyrus (DG, **H**) of wild-type (WT) mice treated with LPS or saline. Scale bar, 50 μm. **B–E**,** I–L**. Quantification of FPR2 fluorescence intensity (**B**,** I**), Manders’ coefficient M1 (the fraction of IBA1 colocalized with FPR2, **C**,** J**), the percentage of FPR2^+^IBA1^+^ cells among IBA1^+^ cells (**D**,** K**), and the area of FPR2 colocalized with IBA1 (**E**,** L**) in the PFC (**B–E**) and hippocampal DG (**I–L**). **F–G**,** M–N**. Line scan analysis of FPR2 and IBA1 fluorescence intensity along a 30 μm segment in the PFC (**F**,** G**) and hippocampal DG (**M**,** N**) of mice treated with saline (WT-Saline) or LPS (WT-LPS). Data are presented as means ± SEM. For each region, 1–2 individual fields were analyzed per mouse, with *n* = 3 mice per group. A two-tailed unpaired Student’s t-test was used to compare two groups. Statistical comparisons between groups are indicated by lines with *p*-values above

Since FPR2 is implicated in LPS-induced acute depressive and anxiety-like behaviors, we next investigated changes in its expression in the brains of LPS-treated mice. Previous studies indicate that FPR2 in the brain is primarily expressed in microglia [18, 19]. Here, our immunofluorescence staining revealed that intraperitoneal LPS injection significantly increased FPR2 expression in the PFC and hippocampus (including the dentate gyrus (DG), cornu ammonis-1 (CA1), and cornu ammonis-3 (CA3)) of mice 24 h post-injection (Fig. 2A, B, H and I, and Additional file 1: Figures S1A, S1B, S1H, S1I). Mander’s analysis showed that in control mice, approximately 60–70% of the total FPR2 fluorescence area colocalized with IBA1 (a microglial marker) in these brain regions (Fig. 2C and J, and Additional file 1: Figures S1C, S1J), indicating that FPR2 is predominantly expressed in microglia under physiological conditions. Following LPS administration, both the number of cells and the area of FPR2 and IBA1 colocalization significantly increased (Fig. 2D, E, K and L, and Additional file 1: Figures S1D, S1E, S1K, S1L), with the colocalized proportion rising to 80–90% of the total FPR2 fluorescence area (Fig. 2C and J, and Additional file 1: Figures S1C, S1J). These findings were corroborated by fluorescence intensity line profile analysis (generated using ImageJ’s Plot Profile tool; Fig. 2F, G, M and N, and Additional file 1: Figures S1F, S1G, S1M, S1N), confirming that intraperitoneal LPS challenge specifically upregulates FPR2 expression in microglia.

Additionally, FPR2 expression has been reported in neurons [18, 19]. Our immunofluorescence staining showed that in the PFC and hippocampus of control mice, a small number of neurons exhibited FPR2-positive staining (Additional file 1: Figures S2A, S2B, S2D, S2E). Furthermore, LPS administration did not significantly alter the proportion of FPR2-positive neurons in these brain regions (Additional file 1: Figures S2A, S2B, S2D, S2E), but it increased FPR2 expression intensity in neurons (Additional file 1: Figures S2C, S2F). However, the magnitude of this increase was much smaller than that observed in microglia (Additional file 1: Figures S2C, S2F). Two-way ANOVA revealed that this cell-type-specific difference was statistically significant in both the PFC (*p* = 0.0001) and the hippocampus (*p* < 0.0001). These results suggest that microglial FPR2 may play a role in LPS-induced depression.

### FPR2 deficiency attenuates LPS-induced microglial activation and neuronal synaptic alterations

**Fig. 3:**
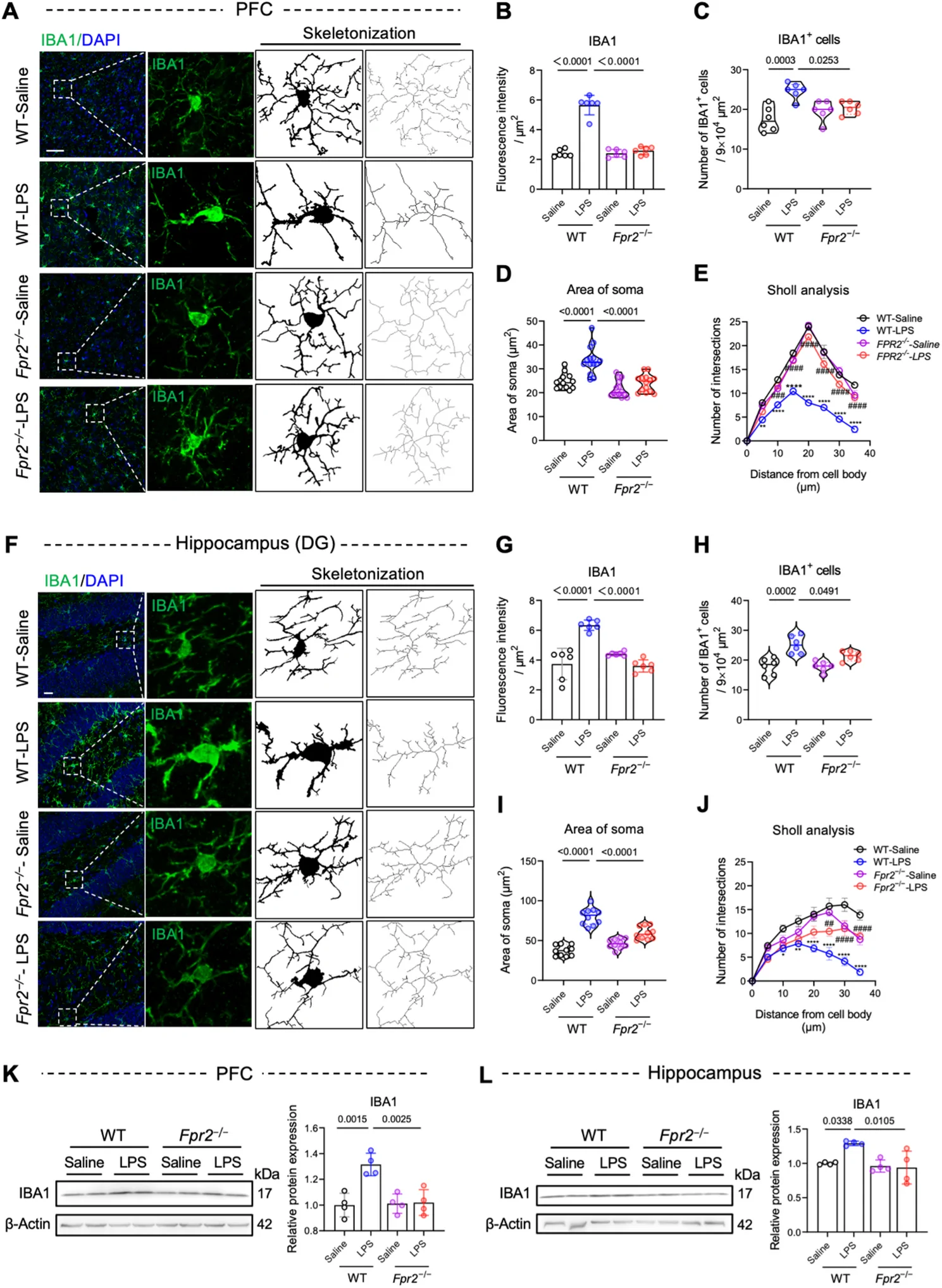
FPR2 deficiency mitigates LPS-induced microglial activation in the PFC and hippocampal DG. **A**,** F**. Representative immunofluorescence and skeletonization images of microglia in the prefrontal cortex (PFC, **A**) and hippocampal dentate gyrus (DG, **F**) of wild-type (WT) and *Fpr2*^–/–^ mice 24 h after LPS or saline administration. Scale bars: 40 μm (**A**), 20 μm (**F**). **B–E**,** G–J**. Quantification of IBA1 fluorescence intensity (**B**,** G**), number of IBA1^+^ cells (**C**,** H**), microglial soma area (**D**,** I**), and Sholl analysis of process intersections (**E**,** J**) in the PFC (**B–E**) and hippocampal DG (**G–J**). **K**,** L**. Western blot analysis of IBA1 expression in the PFC (**K**) and hippocampus (**L**) of WT and *Fpr2*^–/–^ mice 24 h after LPS or saline administration. Data are presented as means ± SEM. For each region, 2 individual fields were analyzed per mouse for IBA1 intensity and cell count (**B–C**,** G–H**); 10–14 cells for soma area (**D**,** I**); 8 cells for Sholl analysis (**E**,** J**); *n* = 3 mice per group for immunofluorescence. For Western blot, *n* = 4 mice per group. One-way ANOVA (B–D, G–I, K–L) and two-way ANOVA (E, J), followed by Tukey’s post hoc test, were used for multiple comparisons. Statistical comparisons between groups are indicated by lines with *p*‑values above. * *p* < 0.05, ** *p* < 0.01, **** *p* < 0.0001 for WT-LPS vs. WT-saline group. ^##^ *p* < 0.01, ^###^ *p* < 0.001, ^####^ *p* < 0.0001 for *Fpr2*^–/–^-LPS vs. WT-LPS group

Given the upregulation of FPR2 in microglia in the LPS-induced model, we investigated the impact of FPR2 deletion on microglial activation. Consistent with previous studies [48], we found that, compared with control mice, LPS administration significantly increased IBA1 fluorescence intensity and the number of IBA1-positive (IBA1^+^) cells in the mouse prefrontal cortex and hippocampus (Fig. 3A–C and F–H, and Additional file 1: Figures S3A–C, S3F–H). This was accompanied by enlarged microglial cell bodies (Fig. 3D and I, and Additional file 1: Figure S3D, S3I) and reduced intersection numbers (Fig. 3E and J, and Additional file 1: Figures S3E, S3J), confirming robust microglial activation. Notably, FPR2 knockout markedly reversed these changes, reducing IBA1 fluorescence intensity and IBA1^+^ cell numbers, decreasing microglial soma size, and increasing intersection numbers (Fig. 3A–J and Additional file 1: Figures S3A–S3J). Furthermore, Western blot analysis confirmed that FPR2 knockout reversed the LPS-induced upregulation of IBA1 protein levels in the PFC and hippocampus (Fig. 3K and L). These results demonstrate that FPR2 deficiency alleviates LPS-induced microglial activation. In addition, correlation analysis revealed a positive correlation between FPR2 and IBA1 expression (Additional file 1: Figure S4). These findings imply a potential role for FPR2 in mediating microglial activation.

**Fig. 4:**
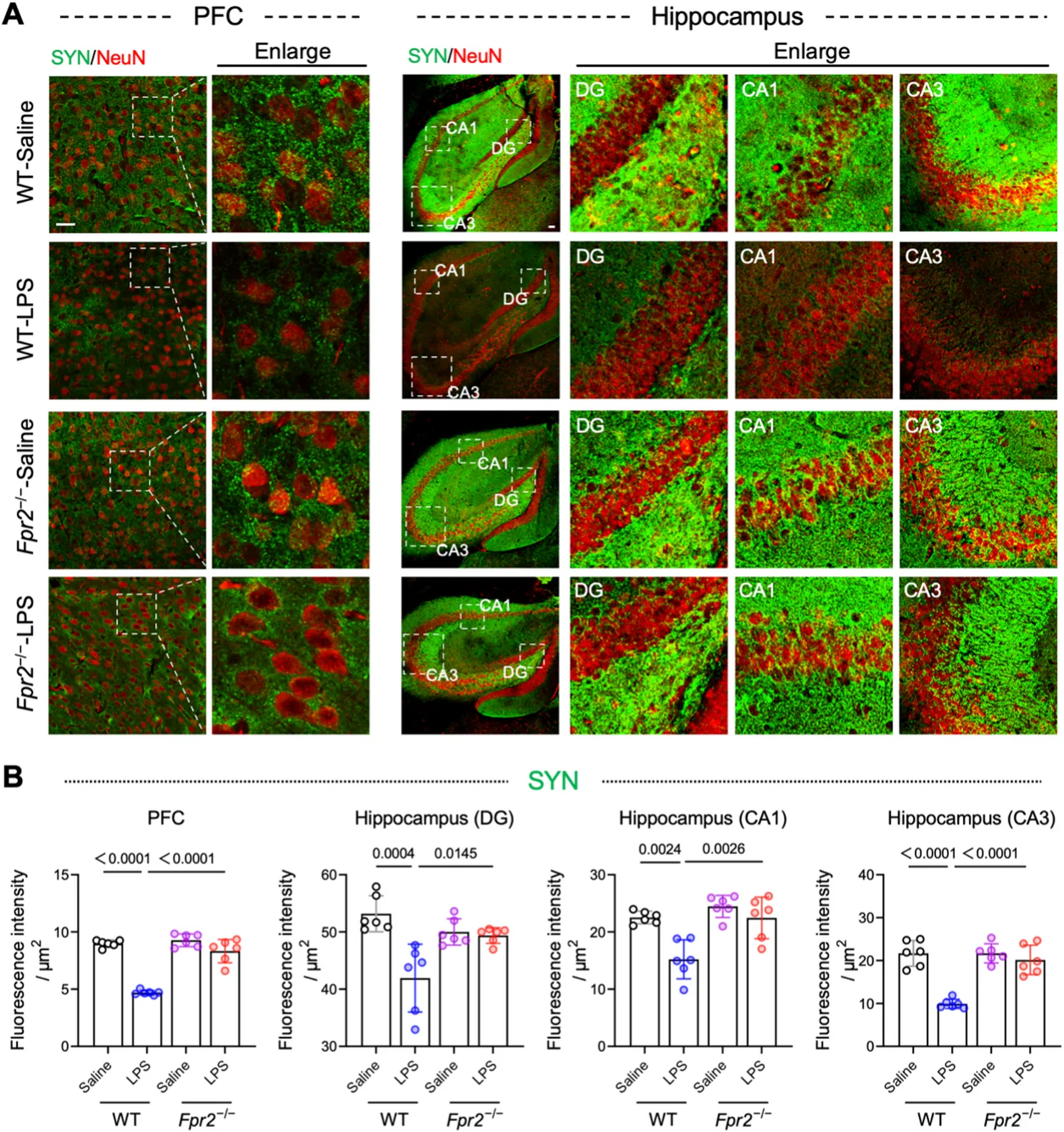
FPR2 deficiency reverses LPS-induced synaptic alterations. (**A**) Representative immunofluorescence images showing presynaptic integrity labeled by synaptophysin (SYN) in the prefrontal cortex (PFC, left) and hippocampus (right) of WT and *Fpr2*^–/–^ mice following LPS or saline administration. Scale bar: 50 μm. (**B**) Quantification of SYN fluorescence intensity in the PFC, hippocampal dentate gyrus (DG), cornu ammonis-1 (CA1), and CA3 regions. Data are presented as mean ± SEM. Two individual fields were analyzed per mouse for each region, with *n =* 3 mice per group. One-way ANOVA followed by Tukey’s post hoc test was used for multiple comparisons. Statistical comparisons between groups are indicated by lines with *p*‑values above

Activated microglia release pro-inflammatory cytokines, triggering neuroinflammation that can lead to neuronal damage [49, 50]. Therefore, we further examined the effect of FPR2 on LPS-induced neuronal injury. Compared with saline-treated controls, LPS challenge significantly downregulated expression of the synaptic vesicle protein synaptophysin (SYN) in the PFC and hippocampus of mice (Fig. 4A and B). This downregulation was reversed by FPR2 knockout (Fig. 4A and B). These results indicate that FPR2 contributes to LPS-induced synaptic alterations and that its deficiency alleviates these changes.

### FPR2 knockout partially rescues LPS-induced alterations in the microglial transcriptome and suppresses activation of the NLRP3 inflammasome signaling pathway

**Fig. 5:**
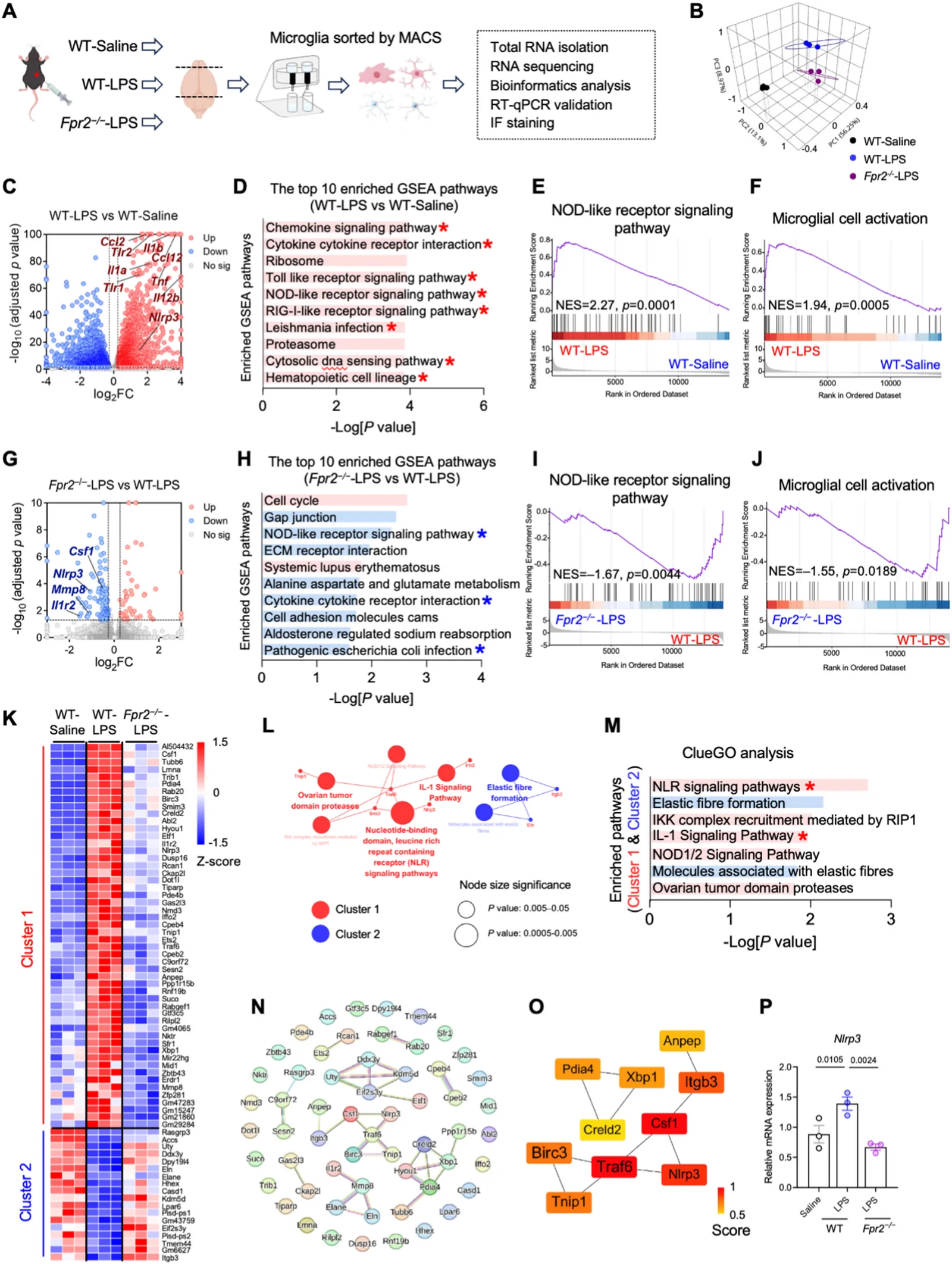
FPR2 deficiency partially rescues LPS-induced alterations in the microglial transcriptome. (**A**) Schematic of the experimental workflow for RNA-seq, bioinformatic analysis, and subsequent validation. (**B**) Three-dimensional principal component analysis (PCA) of microglial transcriptomes. *n* = 3 mice per group. (**C**) Volcano plot depicting differentially expressed genes (DEGs) in WT-LPS vs. WT-Saline microglia. DEGs are defined as adjusted *p* < 0.05 and absolute fold change > 1.2. (**D**) Top 10 enriched pathways identified by gene set enrichment analysis (GSEA) in WT-LPS vs. WT-Saline microglia, ranked by -log₁₀(*p*). Red asterisks indicate immune/inflammatory response-related categories. **E**, **F**. GSEA enrichment plots for the NOD-like receptor (NLR) signaling pathway (**E**) and microglial cell activation pathway (**F**) in WT-LPS vs. WT-Saline microglia. **G**. Volcano plot of DEGs in *Fpr2*^–/–^-LPS vs. WT-LPS microglia, with DEG criteria as in (**C**). **H**. Top 10 enriched pathways identified by GSEA in *Fpr2*^–/–^-LPS vs. WT-LPS microglia, ranked by -log₁₀(*p*). Blue asterisks indicate immune/inflammatory response-related categories. **I**, **J**. GSEA enrichment plots for the NLR signaling pathway (**I**) and microglial cell activation pathway (**J**) in *Fpr2*^–/–^-LPS vs. WT-LPS microglia. **K**. Heatmap of DEGs whose expression was reversed by FPR2 deletion. FPR2 deficiency rescued 52 upregulated (Cluster 1) and 18 downregulated (Cluster 2) DEGs (total 70 genes) in WT-LPS vs. WT-Saline microglia. **L**, **M**. ClueGO analysis of these 70 reversed genes shows enrichment in the NLR signaling pathway and IL-1 signaling pathway. FPR2 knockout suppressed the upregulation of pathway-related genes, including *Nlrp3*, *Traf6*, *Birc3*, and *Il1r2*. **N**, **O**. Protein-protein interaction (PPI) network of the 70 reversed genes, constructed using STRING and analyzed in Cytoscape. The top 10 hub genes were identified by the Radiality method using cytoHubba. **P**. RT-qPCR validation of *Nlrp3* transcript levels in microglia from WT-Saline, WT-LPS, and *Fpr2*^*–/–*^-LPS groups. Data are presented as mean ± SEM, *n* = 3 mice per group. One-way ANOVA followed by Tukey’s post hoc test was used for multiple comparisons. Statistical comparisons between groups are indicated by lines with *p*-values above. NES: normalized enrichment score

To further investigate the mechanism by which FPR2 mediates microglial activation in the LPS-induced depression model, we isolated microglia from WT-Saline, WT-LPS, and *Fpr2*^–/–^-LPS mice for RNA sequencing (Fig. 5A). Three-dimensional principal component analysis (PCA) revealed distinct clustering of the transcriptomic profiles among the three microglial groups (Fig. 5B). Transcriptomic analysis of microglia derived from the WT-Saline and WT-LPS groups identified 6701 differentially expressed genes (DEGs; adjusted *p* < 0.05, absolute fold change |FC| > 1.2), comprising 2,927 upregulated and 3,774 downregulated genes (Fig. 5C). Ranking DEGs by statistical significance revealed that many of the top 50 genes were immune-related and upregulated, including *Il1b*, *Il1a*, *Il12b*, *Ccl2*, *Ccl12*, *Tlr2*, *Tlr1*, and *Tnf* (Fig. 5C and Additional file 2: Table S6). Consistent with immunofluorescence results (Fig. 2 and Additional file 1: Figure S1), LPS challenge significantly elevated *Fpr2* transcript levels in microglia (adjusted *p* = 6.75 × 10^− 23^, FC = 6.63, Additional file 2: Table S6). Gene set enrichment analysis (GSEA) further showed that the top 10 enriched pathways in WT-LPS microglia were all upregulated compared to WT-Saline controls (Fig. 5D). Notably, eight of these pathways were associated with immune or inflammatory responses, including the chemokine signaling pathway, cytokine-cytokine receptor interaction, toll-like receptor signaling pathway, NOD-like receptor (NLR) signaling pathway, and RIG-I-like receptor signaling pathway (Fig. 5D and E; red asterisks). In addition, the microglial cell activation pathway was significantly enriched (Fig. 5F). Together, these findings indicate that LPS triggers microglial activation and enhances immune reactivity in the mouse brain.

In contrast to WT-LPS mice, FPR2 knockout significantly altered the transcription of 153 genes (adjusted *p* < 0.05, |FC| > 1.2) in microglia from *Fpr2*^–/–^-LPS mice, including 46 upregulated and 107 downregulated genes (Fig. 5G and Additional file 3: Table S7). Several immune-related genes, including *Csf1*, *Nlrp3*, *Mmp8*, and *Il1r2*, were downregulated among the DEGs. GSEA showed that, compared to WT-LPS microglia, eight of the top ten enriched pathways in *Fpr2*^–/–^-LPS mice were downregulated, including the NLR signaling pathway, cytokine–cytokine receptor interaction, and pathogenic Escherichia coli infection pathway (Fig. 5H, blue asterisks). Notably, FPR2 knockout reversed the LPS-induced increase in the NLR signaling pathway in microglia (Fig. 5I). Additionally, FPR2 deletion reduced the upregulation of the microglial cell activation pathway (Fig. 5J). We further analyzed DEGs whose transcriptional levels were reversed by FPR2 deletion. Specifically, FPR2 knockout rescued 52 upregulated (Fig. 5K; Cluster 1) and 18 downregulated (Fig. 5K; Cluster 2) DEGs (70 genes in total) identified in the WT-LPS versus WT-Saline comparison. ClueGO analysis of these reversed genes revealed enrichment in the NLR signaling pathway and its downstream IL-1 signaling pathway (Fig. 5L and M). Consistently, FPR2 knockout significantly suppressed the upregulation of pathway-related genes, including *Nlrp3*, *Traf6*, *Birc3*, and *Il1r2* (Fig. 5L). Protein-protein interaction (PPI) network analysis of the 70 reversed genes was performed using the STRING database and analyzed in Cytoscape with cytoHubba and CytoNCA. Among the top 10 hub genes identified, *Csf1*, *Traf6*, and *Nlrp3* ranked as the top three (Fig. 5N and O, and Additional file 1: Tables S2, S3). These results demonstrate that FPR2 deletion reverses LPS-induced microglial activation and restores transcriptomic changes associated with immune-inflammatory signaling, particularly genes related to the NLRP3 pathway.

**Fig. 6:**
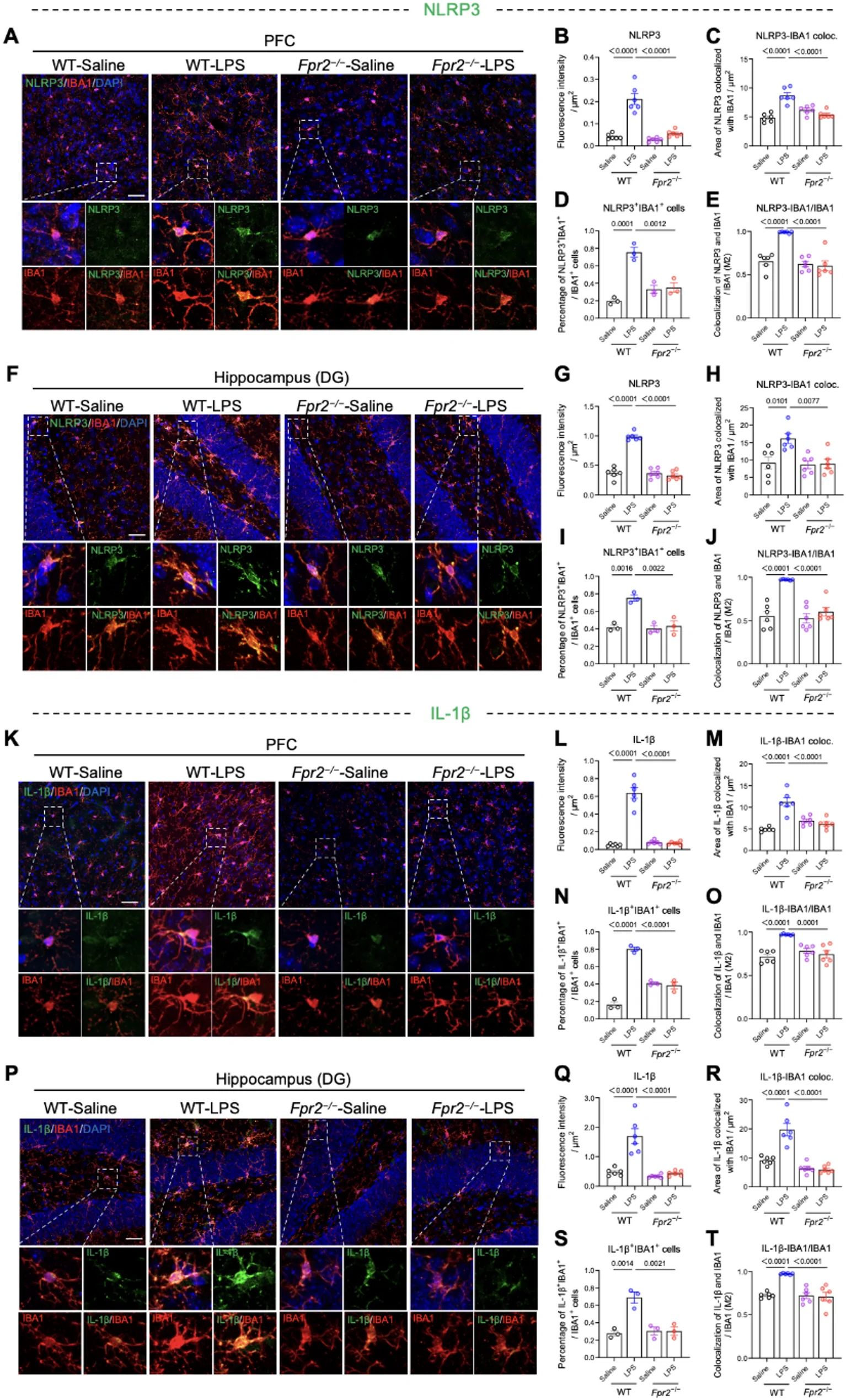
FPR2 deficiency suppresses activation of the NLRP3 inflammasome signaling pathway in the PFC and hippocampal DG. **A**, **F**. Representative immunofluorescence images showing NLRP3 expression in the prefrontal cortex (PFC, **A**) and hippocampal dentate gyrus (DG, **F**) of WT and *Fpr2*^–/–^ mice after LPS or saline administration. Scale bar: 40 μm. **B**–**E**, **G**–**J**. Quantification of NLRP3 fluorescence intensity (**B**, **G**), the area of NLRP3 colocalized with IBA1 (**C**, **H**), the percentage of NLRP3⁺IBA1⁺ cells among IBA1^+^ cells (**D**, **I**), and Manders’ coefficient M2 (the fraction of NLRP3 colocalized with IBA1, **E**, **J**) in the PFC (**B**–**E**) and hippocampal DG (**G**–**J**). K, P. Representative immunofluorescence images showing IL-1β expression in the PFC (**K**) and hippocampal DG (**P**) of WT and *Fpr2*^–/–^ mice after LPS or saline administration. **L**–**O**, **Q**–**T**. Quantification of IL-1β fluorescence intensity (**L**,** Q**), the area of IL-1β colocalized with IBA1 (**M**,** R**), the percentage of IL-1β⁺IBA1⁺ cells among IBA1^+^ cells (**N**,** S**), and Manders’ coefficient M2 (the fraction of IL-1β colocalized with IBA1, **O**,** T**) in the PFC (**L–O**) and hippocampal DG (**Q–T**). Data are presented as means ± SEM. For each region, 1–2 individual fields were analyzed per mouse, with *n* = 3 mice per group. One-way ANOVA followed by Tukey’s post hoc test was used for multiple comparisons. Statistical comparisons between groups are indicated by lines with *p*‑values above

We next validated NLRP3 expression using RT‑qPCR and immunofluorescence staining. Consistent with the RNA-seq results, RT‑qPCR confirmed that LPS challenge upregulated *Nlrp3* transcription in microglia in the mouse brain compared with WT-Saline mice (Fig. 5P). Immunofluorescence staining showed that LPS treatment significantly increased NLRP3 fluorescence intensity in the PFC and hippocampus (Fig. 6A, B, F and G, and Additional file 1: Figures S5A, S5B, S5F, S5G), and enhanced both the area of NLRP3 and IBA1 colocalization and the number of colocalized cells (Fig. 6C, D, H and I, and Additional file 1: Figures S5C, S5D, S5H, S5I). Notably, the proportion of NLRP3 fluorescence area that colocalized with IBA1 increased significantly to nearly 100% (Fig. 6E and J, and Additional file 1: Figures S5E, S5J), indicating that LPS-induced upregulation of NLRP3 occurs predominantly in microglia. As expected, FPR2 knockout markedly reversed these LPS-induced changes (Fig. 6A–J and Additional file 1: Figures S5A–S5J). We then examined IL-1β expression, a downstream effector of the NLRP3 signaling pathway. In line with the changes in NLRP3, FPR2 deletion suppressed LPS-induced upregulation of *Il1b* transcription (Additional file 1: Figure S6), reduced IL‑1β fluorescence intensity in the PFC and hippocampus, and decreased both the area of IL‑1β and IBA1 colocalization and the number of colocalized cells (Fig. 6K–N and P–S, and Additional file 1: Figures S5K–S5N, S5P–S5S). Moreover, the proportion of IL‑1β fluorescence area that colocalized with IBA1 also decreased (Fig. 6O and T, and Additional file 1: Figures S5O, S5T). Collectively, these data confirm that FPR2 deficiency attenuates LPS-induced activation of the NLRP3 pathway in microglia.

### FPR2 mediates LPS-induced activation of the NLRP3 pathway in microglia through CSF1

**Fig. 7:**
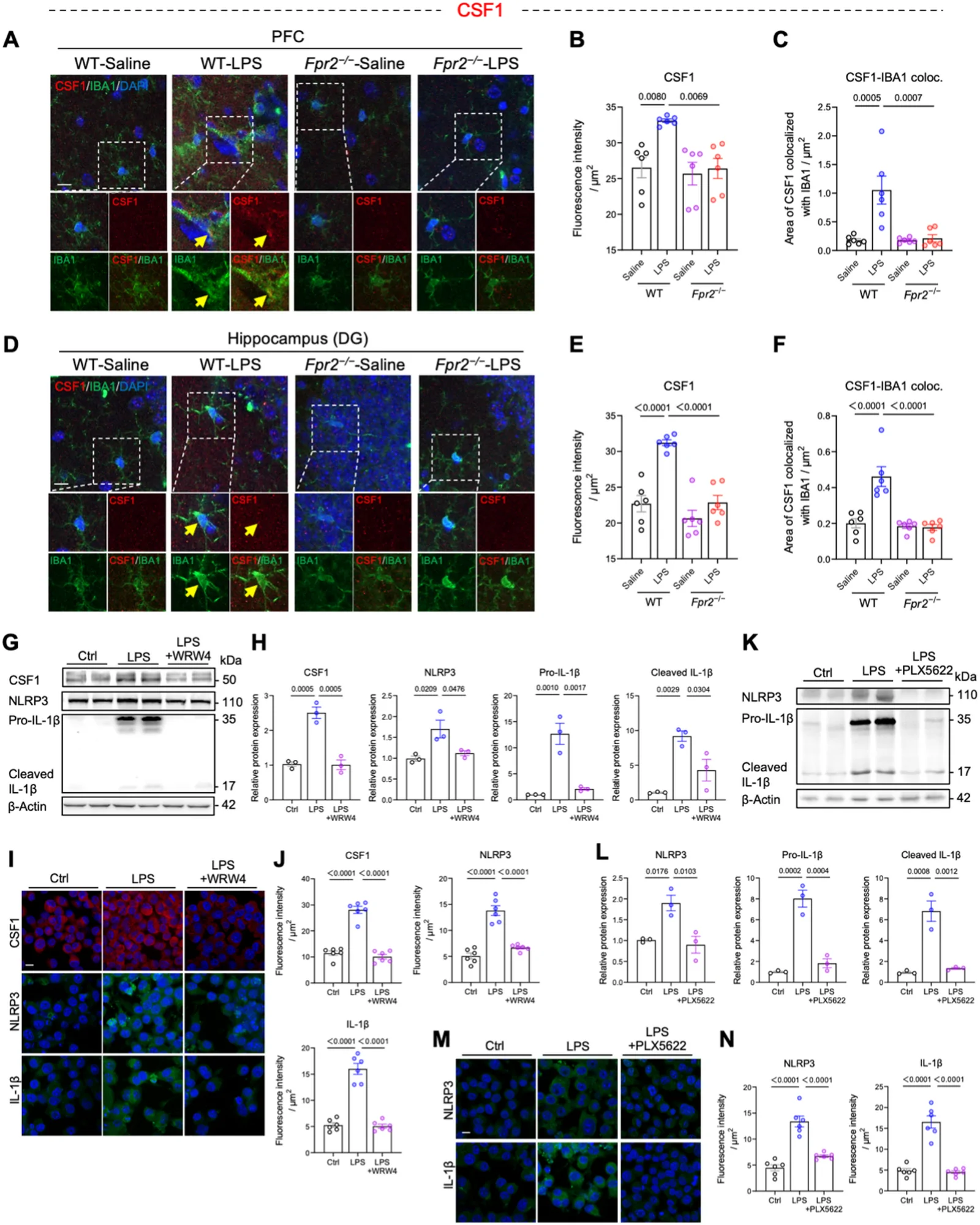
FPR2 mediates LPS-induced activation of the NLRP3 inflammasome pathway in microglia via CSF1. **A–C**. Representative immunofluorescence images (**A**) and quantification (**B**,** C**) of CSF1 expression and colocalization with IBA1 in the prefrontal cortex (PFC) of WT or *Fpr2*^–/–^ mice after LPS or saline administration. Scale bar: 10 μm. **D–F**. Representative immunofluorescence images (**D**) and quantification (**E**,** F**) of CSF1 expression and colocalization with IBA1 in the hippocampal dentate gyrus (DG) of WT or *Fpr2*^–/–^ mice after LPS or saline administration. Scale bar: 10 μm. **G**,** H**. Western blot analysis (**G**) and quantification (**H**) of CSF1, NLRP3, pro-IL-1β, and cleaved IL-1β levels in N9 microglial cells treated with media alone (control, Ctrl), 1 µg/mL LPS, or 1 µg/mL LPS with 10 µM WRW4. **I**,** J**. Representative immunofluorescence images (**I**) and quantification (**J**) of CSF1, NLRP3, and IL-1β expression in N9 cells treated with media alone, 1 µg/mL LPS, or 1 µg/mL LPS with 10 µM WRW4. Scale bar: 10 μm. **K**,** L**. Western blot analysis (**K**) and quantification (**L**) of NLRP3, pro-IL-1β, and cleaved IL-1β levels in N9 cells treated with media alone, 1 µg/mL LPS, or 1 µg/mL LPS with 1 µM PLX5622. **M**,** N**. Representative immunofluorescence images (**M**) and quantification (**N**) of NLRP3 and IL-1β expression in N9 microglia treated with media alone, 1 µg/mL LPS, or 1 µg/mL LPS with 1 µM PLX5622. Scale bar: 10 μm. Data are presented as means ± SEM. For in vivo experiments (**A–F**), two individual fields were analyzed per mouse, with *n* = 3 mice per group. For in vitro experiments (**G–N**), *n* = 3 independent cell cultures per group. One-way ANOVA followed by Tukey’s post hoc test was used for multiple comparisons. Statistical comparisons between groups are indicated by lines with *p*‑values above

Our RNA-seq data showed that FPR2 deficiency significantly suppressed LPS-induced upregulation of CSF1 in mouse microglia (Fig. 5K and O). Previous studies indicate that FPR2 participates in regulating the CSF1 signaling pathway [51, 52], and inhibition of the CSF1 receptor (CSF1R) has been reported to suppress NLRP3 pathway activation [53]. We therefore hypothesized that FPR2 might regulate the NLRP3 signaling pathway in microglia through the CSF1/CSF1R axis. We first validated the RNA-seq findings by examining CSF1 protein levels in microglia using immunofluorescence staining. LPS challenge increased both overall CSF1 levels and CSF1 expression specifically in microglia in the prefrontal cortex and hippocampus compared with WT-Saline mice (Fig. 7A–F and Additional file 1: Figures S7A–S7F). These changes were reversed by FPR2 knockout (Fig. 7A–F and Additional file 1: Figures S7A–S7F), indicating that FPR2 contributes to LPS-induced upregulation of CSF1 in microglia.

To further investigate how FPR2 regulates CSF1 and the NLRP3 pathway, we performed in vitro experiments using the mouse N9 microglial cell line. Western blot and immunofluorescence assays showed that LPS (1 µg/mL) significantly increased expression of CSF1, NLRP3, and IL-1β in N9 cells, whereas treatment with the FPR2 antagonist WRW4 suppressed this upregulation (Fig. 7G–J). Moreover, administration of the CSF1R inhibitor PLX5622 reversed the LPS-induced increase in NLRP3 and IL-1β expression (Fig. 7K–N). These findings suggest that both FPR2 and CSF1 are involved in LPS-induced activation of the NLRP3 pathway.

**Fig. 8:**
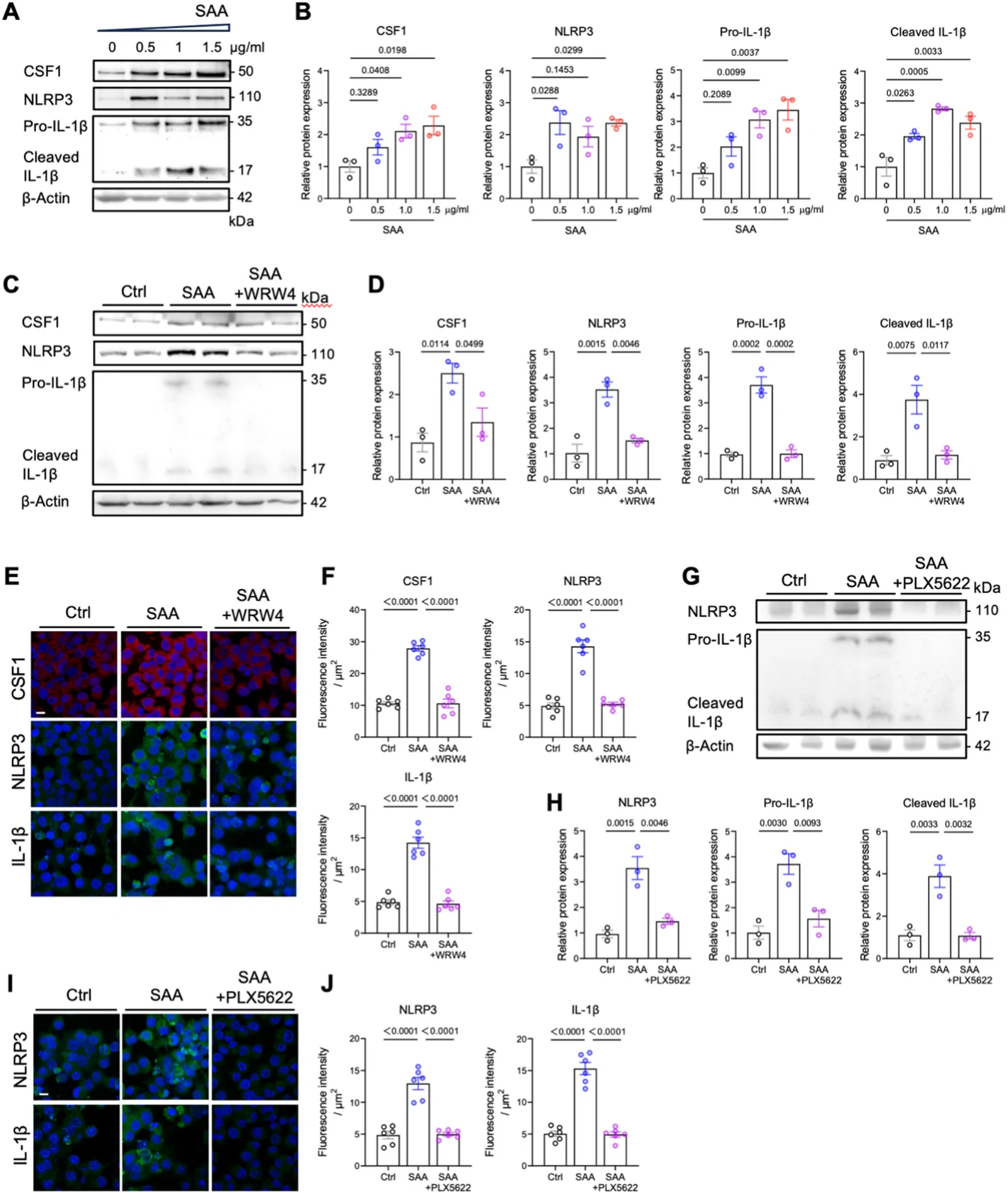
FPR2 mediates SAA-induced activation of the NLRP3 inflammasome pathway in microglia via CSF1. **A**,** B**. Western blot analysis (**A**) and quantification (**B**) of CSF1, NLRP3, pro-IL-1β, and cleaved IL-1β in N9 microglial cells treated with SAA (0–1.5 µg/ml). **C**,** D**. Western blot analysis (**C**) and quantification (**D**) of CSF1, NLRP3, pro-IL-1β, and cleaved IL-1β in N9 cells treated with media alone (control, Ctrl), 1.5 µg/ml SAA, or 1.5 µg/ml SAA with 10 µM WRW4. **E**,** F**. Representative immunofluorescence images (**E**) and quantification (**F**) of CSF1, NLRP3, and IL-1β expression in N9 cells treated with media alone, 1.5 µg/ml SAA, or 1.5 µg/ml SAA with 10 µM WRW4. Scale bar: 10 μm. **G**,** H**. Western blot analysis (**G**) and quantification (**H**) of NLRP3, pro-IL-1β, and cleaved IL-1β in N9 cells treated with media alone, 1.5 µg/ml SAA, or 1.5 µg/ml SAA with 1 µM PLX5622. **I**,** J**. Representative immunofluorescence images (**I**) and quantification (**J**) of NLRP3 and IL-1β expression in N9 cells treated with media alone, 1.5 µg/ml SAA, or 1.5 µg/ml SAA with 1 µM PLX5622. Scale bar: 10 μm. Data are presented as means ± SEM. *n* = 3 independent cell cultures per group. One-way ANOVA followed by Tukey’s post hoc test was used for multiple comparisons. Statistical comparisons between groups are indicated by lines with *p*‑values above. SAA: recombinant human serum amyloid A 1/2 (rh-SAA)

To determine whether FPR2 functions upstream of CSF1, we treated N9 cells with a commercially available FPR2 agonist, recombinant human SAA (rh-SAA; a consensus molecule combining sequence features of both SAA1 and SAA2 isoforms), which also activates murine FPR2 [54]. SAA1 and SAA2 are functional FPR2 ligands known to induce immune cell activation and proinflammatory responses [55]. As with LPS (1 µg/mL) treatment, rh-SAA significantly increased the expression of CSF1, NLRP3, and IL-1β (Fig. 8A and B). This induction was inhibited by the FPR2 antagonist WRW4 (Fig. 8C–F) and attenuated by the CSF1R inhibitor PLX5622 (Fig. 8G–J). These results indicate that FPR2 and CSF1 are involved in SAA-induced activation of the NLRP3 pathway, with FPR2 acting upstream of CSF1 to mediate this response.

Notably, commercial rh-SAA preparations may contain trace amounts of endotoxin contamination (0.1 ng LPS per 1 µg SAA). To rule out the possibility that the observed effects were attributable to LPS contamination, we treated N9 cells with 2.5 ng/ml LPS, a concentration approximately 16.7-fold higher than the estimated LPS content of 1.5 µg/ml rh-SAA (the highest concentration used in our experiments). This LPS treatment alone (2.5 ng/ml) had no effect on CSF1, NLRP3, or IL-1β expression (Additional file 1: Figure S8), confirming that the effects of rh-SAA were not confounded by endotoxin contamination.

### The SAA/FPR2 axis contributes to depressive and anxiety-like behaviors in mice via CSF1

**Fig. 9:**
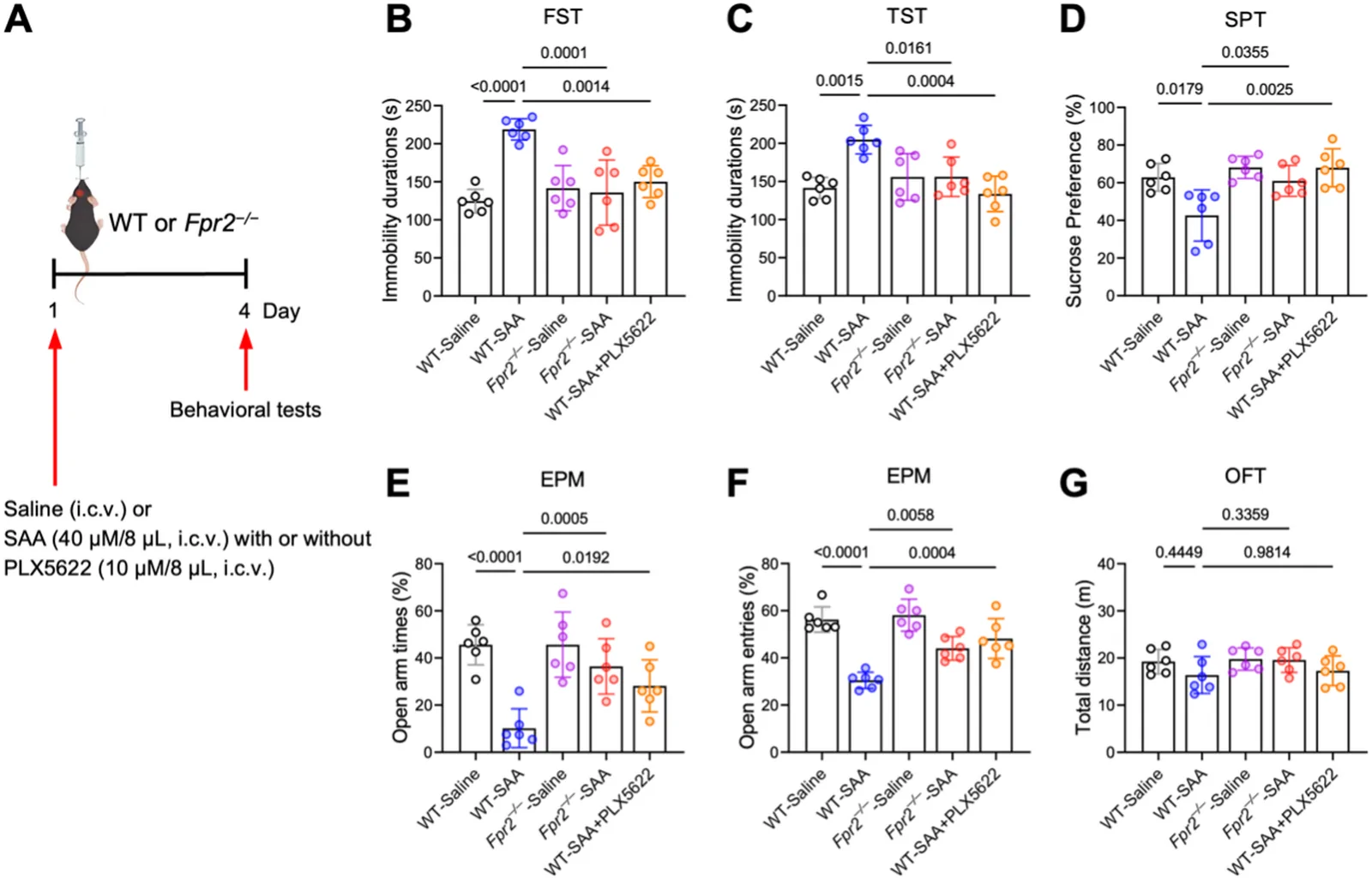
The SAA/FPR2 axis contributes to depressive and anxiety-like behaviors in mice via CSF1. **A**. Schematic of the experimental procedure for intracerebroventricular (i.c.v.) administration of SAA, with or without PLX5622, in wild-type (WT) and *Fpr2*^–/–^ mice. **B–C**. Immobility time in the forced swim test (FST, **B**) and tail suspension test (TST, **C**) of WT and *Fpr2*^–/–^ mice after i.c.v. administration of saline, SAA, or SAA with PLX5622. **D**. Sucrose preference in the sucrose preference test (SPT) of WT and *Fpr2*^–/–^ mice after i.c.v. administration of saline, SAA, or SAA with PLX5622. **E–F**. Percentage of time spent on open arms (**E**) and number of open-arm entries (**F**) in the elevated plus maze (EPM) test of WT and *Fpr2*^–/–^ mice after i.c.v. administration of saline, SAA, or SAA with PLX5622. **G**. Total distance traveled in the open field test (OFT) of WT and *Fpr2*^–/–^ mice after i.c.v. administration of saline, SAA, or SAA with PLX5622. Data are presented as means ± SEM. *n* = 6 mice per group. One-way ANOVA followed by Tukey’s post hoc test was used for multiple comparisons. Statistical comparisons between groups are indicated by lines with *p*‑values above. SAA: recombinant human serum amyloid A 1/2 (rh-SAA)

Given that our findings demonstrate that FPR2 regulates NLRP3 activation via CSF1, we speculate that FPR2 may contribute to depressive and anxiety-like behaviors in mice through the CSF1/NLRP3 axis. We first administered rh-SAA to mice via intracerebroventricular (ICV) injection to examine whether SAA could induce depressive and anxiety-like behaviors by activating FPR2 (Fig. 9A). Compared with control mice, ICV administration of rh-SAA significantly increased immobility time in the FST and TST (Fig. 9B and C), decreased sucrose preference in the SPT (Fig. 9D), and reduced time spent and the number of entries into the open arms in the EPM (Fig. 9E and F). However, these behavioral abnormalities were not observed in *Fpr2*^–/–^ mice after ICV injection of rh-SAA (Fig. 9B–F). These findings suggest that SAA induces depressive and anxiety-like behaviors specifically through FPR2.

We next investigated whether FPR2 participates in these SAA-induced abnormal behaviors through the CSF1/CSF1R axis. We found that ICV administration of the CSF1R inhibitor PLX5622 significantly ameliorated rh-SAA-induced depressive and anxiety-like behaviors (Fig. 9B–F), indicating that the SAA/FPR2 axis promotes these behaviors by activating the CSF1/CSF1R pathway. Moreover, no significant differences were observed in total distance traveled in the OFT (Fig. 9G), indicating that ICV administration of rh-SAA, with or without PLX5622, did not affect general locomotor activity.

**Fig. 10:**
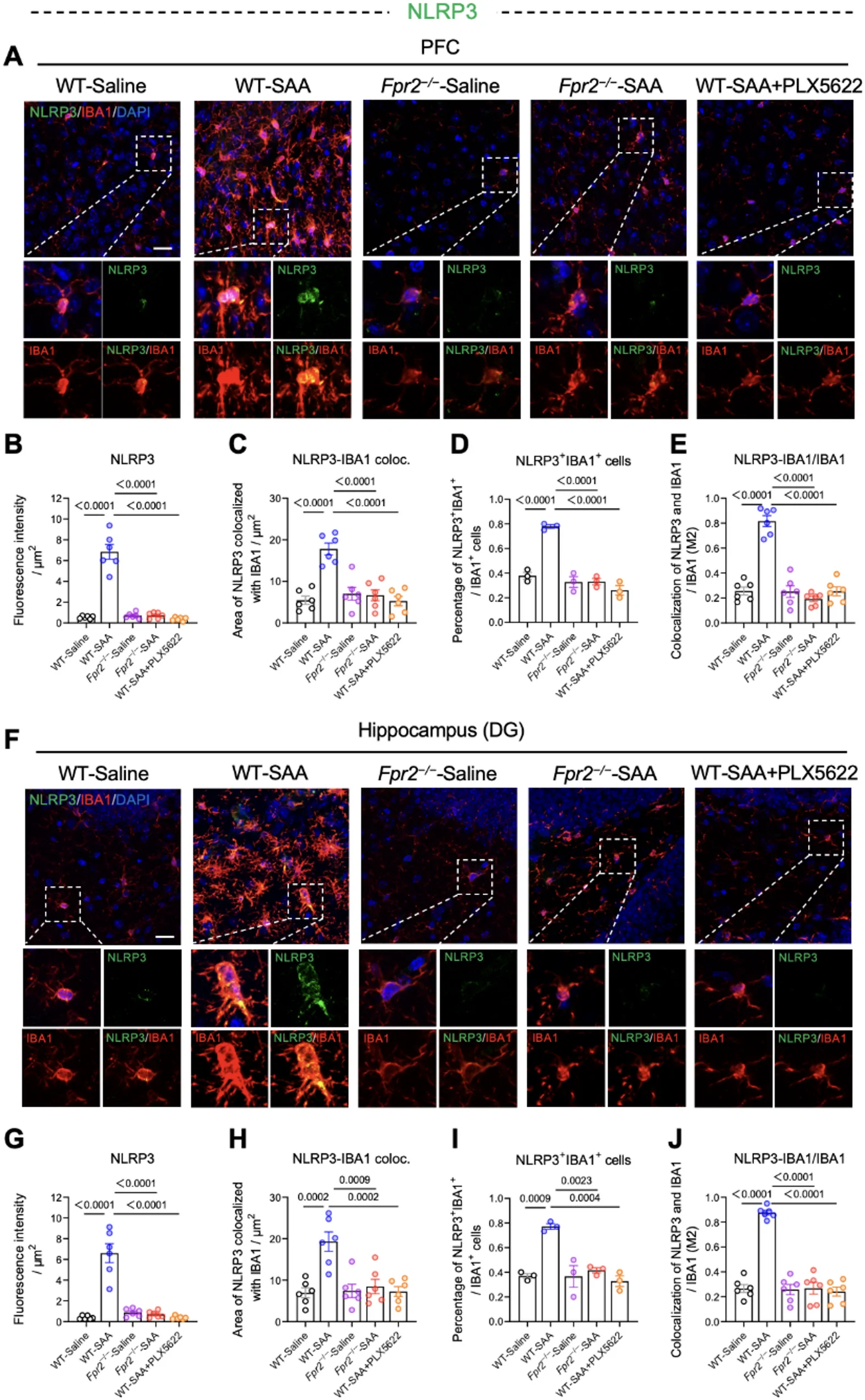
SAA activates NLRP3 in microglia via FPR2 and CSF1R in the PFC and hippocampal DG. **A**,** F**. Representative immunofluorescence images showing NLRP3 expression in the prefrontal cortex (PFC, **A**) and hippocampal dentate gyrus (DG, **F**) of WT and *Fpr2*^–/–^ mice after ICV injection of saline, rh-SAA, or rh-SAA with PLX5622. Scale bar: 10 μm. **B–E**, **G–J**. Quantification of NLRP3 fluorescence intensity (**B**,** G**), the area of NLRP3 colocalized with IBA1 (**C**,** H**), the percentage of NLRP3⁺IBA1⁺ cells among IBA1⁺ cells (**D**,** I**), and Manders’ coefficient M2 (the fraction of NLRP3 colocalized with IBA1, **E**,** J**) in the PFC (**B–E**) and hippocampal DG (**G–J**). Data are presented as means ± SEM. For each region, 1–2 individual fields were analyzed per mouse, with *n* = 3 mice per group. One-way ANOVA followed by Tukey’s post hoc test was used for multiple comparisons. Statistical comparisons between groups are indicated by lines with *p*-values above. SAA: recombinant human serum amyloid A 1/2 (rh-SAA)

To further determine whether SAA activates NLRP3 in microglia via FPR2 and CSF1R, we performed immunofluorescence staining for IBA1 and NLRP3 in brain sections from mice subjected to ICV injection of rh-SAA (Fig. 9A). ICV administration of rh-SAA significantly increased NLRP3 fluorescence intensity in the PFC and hippocampus compared with control mice (Fig. 10A, B, F and G, and Additional file 1: Figures S9A, S9B, S9F, S9G). It also enhanced both the area of NLRP3 and IBA1 colocalization and the number of colocalized cells (Fig. 10C, D, H and I, and Additional file 1: Figures S9C, S9D, S9H, S9I). Moreover, the proportion of NLRP3 fluorescence area that colocalized with IBA1 increased significantly to more than 80% in rh-SAA-treated mice (Fig. 10E and J, and Additional file 1: Figures S9E, S9J), suggesting that SAA-induced NLRP3 upregulation occurs specifically in microglia. However, these rh-SAA-induced changes were absent in *Fpr2*^–/–^ mice (Fig. 10A–J and Additional file 1: Figures S9A–S9J). Furthermore, ICV co-administration of the CSF1R inhibitor PLX5622 completely reversed the rh-SAA-induced increase in NLRP3 expression and microglial NLRP3-IBA1 colocalization (Fig. 10A–J and Additional file 1: Figures S9A–S9J). These results demonstrate that SAA activates the NLRP3 inflammasome in microglia via the FPR2-CSF1/CSF1R axis, consistent with our previous findings in the LPS model.

Additionally, we confirmed that intraperitoneal LPS injection upregulates both the transcriptional and protein levels of SAA1 and SAA2 in the mouse brain (Additional file 1: Figure S10). These findings suggest that LPS may induce depressive and anxiety-like behaviors in mice by stimulating SAA1/SAA2 production, which then acts on microglial FPR2 to activate the downstream CSF1/NLRP3 pathway.

### SAA is upregulated in the ovPFC and in the plasma of patients with MDD

**Fig. 11:**
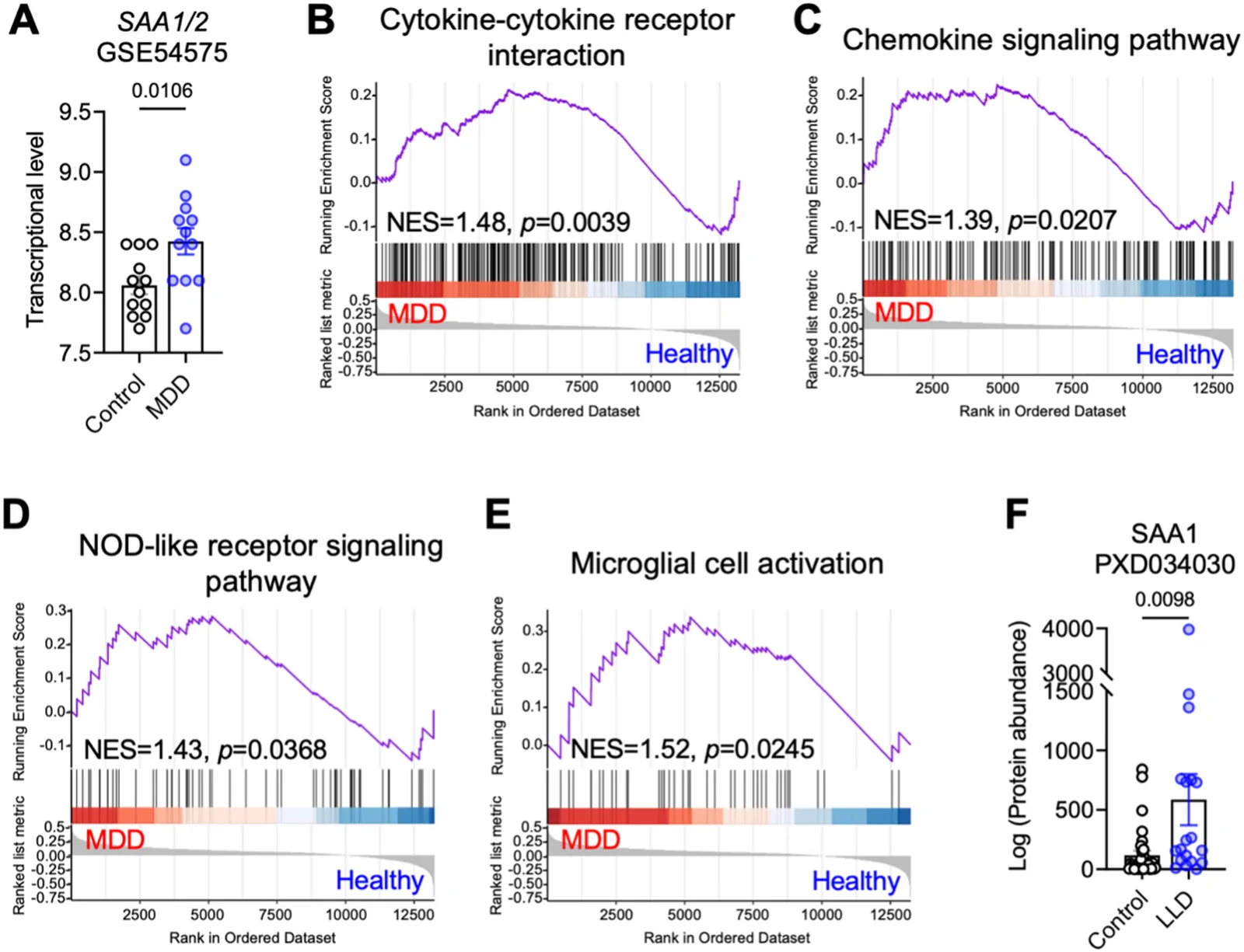
SAA is upregulated in the ovPFC and plasma of patients with major depressive disorder. **A**. Transcript levels of *SAA1/2* in the ovPFC of patients with major depressive disorder (MDD) and healthy controls (dataset GSE54575). **B–E**. Gene set enrichment analysis (GSEA) of dataset GSE54575 shows upregulation of pathways related to cytokine-cytokine receptor interaction (**B**), chemokine signaling (**C**), NOD-like receptor (NLR) signaling (**D**), and microglial cell activation (**E**) in the ovPFC of MDD patients. **F**. Protein abundance of SAA1 in the plasma of late-life depression (LLD) patients and healthy controls (dataset PXD034030). Statistical comparisons between groups are indicated by lines with *p*‑values above. NES: normalized enrichment score

Our study demonstrated that LPS increased SAA1/2 expression in the mouse brain and that intracerebral administration of rh-SAA induces depressive and anxiety-like behaviors in mice via FPR2, suggesting a potential role for SAA in the pathogenesis of MDD. Therefore, we analyzed changes in *SAA1/2* transcriptional levels in brain tissues from MDD patients using data from the NCBI GEO database. Our previous study identified 14 transcriptomic datasets from different brain regions of MDD patients in the NCBI GEO database [19]. Among these, the GSE54575 dataset showed upregulation of FPR2 transcript levels in the orbital ventral PFC (ovPFC) of MDD patients [19]. In this study, we found that *SAA1* expression was significantly upregulated only in this dataset (Fig. 11A). Further GSEA analysis revealed significant upregulation of immune-related pathways, including cytokine-cytokine receptor interaction, chemokine signaling pathway, and NLR signaling pathway (Fig. 11B–D). Additionally, the microglial activation pathway was significantly upregulated (Fig. 11E). These results suggest that in the ovPFC of MDD patients, the SAA/FPR2 axis is enhanced and that microglial activation and immune responses are heightened.

Peripheral administration of LPS has been reported to significantly induce SAA1/2 production in the liver [56], and SAA1/2 can enter the blood and cross the blood-brain barrier [57]. Therefore, we speculate that LPS-induced increases in peripheral SAA1/2 may also contribute to depressive and anxiety-like behaviors by entering the brain. Epidemiological studies have shown elevated serum levels of SAA1 in unmedicated patients with MDD [58, 59]. Furthermore, a search of the ProteomeXchange public proteomics database using the keywords “Depression”, “Major depressive disorder”, or “MDD” and “Homo sapiens” identified three datasets that provided raw proteomic data from healthy controls and medication-naïve depressive patients, or those who had been free of antidepressant or antipsychotic drugs for at least three months before sampling. The accession numbers for these datasets are PXD034030, PXD047302, and PXD050851 (Additional file 1: Table S5). Among them, PXD034030 includes patients with late-life depression (LLD) with mild anxiety symptoms [44], PXD047302 comprises individuals with vascular depression (VaD) [45], and PXD050851 covers patients with postpartum depression (PPD) [46]. Analysis of these datasets revealed elevated plasma SAA1 levels in LLD patients compared with healthy controls (Fig. 11F and Additional file 1: Figure S11). Further GO biological process term analysis of this dataset showed that among the top 10 terms ranked by *p*-value, eight were associated with immune or inflammatory responses (Additional file 1: Figure S12; asterisks) [44]. Enriched terms involving SAA1 included regulation of response to external stimulus, acute inflammatory response, response to stress, and wound healing. These findings suggest that SAA may not only serve as a potential biomarker for depression, especially LLD, but also that increased peripheral SAA, which may ultimately enter the brain, could contribute to the pathogenesis of depression and anxiety disorders.

## Discussion

The present study demonstrates that FPR2 deficiency significantly attenuates depressive and anxiety-like behaviors induced by acute inflammation (i.e., peripheral LPS administration). Our previous study revealed that both peripheral and central administration of the FPR2 antagonist WRW4 alleviates social isolation-induced depressive and anxiety-like behaviors [19]. Furthermore, intracerebroventricular (ICV) injection of resolvin D1 (RvD1), an FPR2 pro-resolving ligand, has been shown to reverse depressive-like behaviors caused by chronic unpredictable stress and LPS in mice [31, 32]. Additionally, FPR2/3 knockout mitigates chronic corticosterone-induced depressive-like behaviors and cognitive impairment [30]. These findings indicate that genetic knockout or pharmacological targeting of FPR2 can alleviate depressive-like symptoms in animal models with various causes. Precise modulation of FPR2 may provide a promising strategy for the treatment of depression.

We further identify the SAA/FPR2/CSF1/NLRP3 axis as a potential regulatory pathway mediating inflammatory depression, elucidating the upstream and downstream molecular mechanisms through which FPR2 regulates microglial activation and neuroinflammation. Our results show that in the brains of LPS-induced depressive mice, FPR2 is significantly upregulated, predominantly in microglia, and that FPR2 deficiency markedly suppresses NLRP3 inflammasome activation and IL-1β release in microglia. Consistent with this, our previous study found that FPR2 is also predominantly upregulated in microglia in social isolation-induced depressive mice [19]. These findings suggest that microglia are the major effector cells mediating FPR2-dependent depressive pathology. Importantly, the SAA-FPR2-CSF1 axis likely interferes primarily with the priming phase of NLRP3 inflammasome activation, the NF-κB-dependent transcriptional upregulation of *Nlrp3* and *Il1b*, rather than directly affecting the assembly of the inflammasome complex. This speculation is consistent with our observation that FPR2 deletion reduces the expression levels of these priming components, whereas we did not directly assess downstream assembly steps. Mechanistically, in vivo and in vitro experiments confirm that FPR2, as an upstream receptor, enhances activation of the downstream NLRP3 inflammasome pathway by regulating CSF1 expression. This aligns with previous studies showing that FPR2 can modulate the CSF1 signaling pathway [51, 52], and that CSF1R inhibitors can block NLRP3 pathway activation [53]. Meanwhile, this study identifies SAA as a key upstream ligand that mediates activation of the FPR2/CSF1/NLRP3 axis. ICV injection of SAA specifically induces depressive-like behaviors in mice via FPR2, and this effect depends on CSF1R activation. As a functional ligand of FPR2 [55], our findings highlight its novel role in the CNS in regulating emotional behavior by activating microglial pro-inflammatory signaling pathways. Furthermore, RNA-seq and validation experiments reveal that FPR2 deletion reverses LPS-induced transcriptomic changes in microglial immune and inflammatory responses, with enrichment in the NLR signaling pathway, further confirming the central regulatory role of this axis in microglial activation and neuroinflammation.

Our results also provide clinical evidence for the involvement of the SAA/FPR2/CSF1/NLRP3 axis in the pathophysiology of MDD, offering potential directions for research on MDD biomarkers and targeted therapy. The present study and our previous work [19] found that the transcriptional levels of *SAA1* and *FPR2* are significantly upregulated in the ovPFC of patients with MDD, and that plasma SAA1 protein levels are elevated in patients with late-life depression. These results are consistent with previous epidemiological studies showing increased peripheral blood SAA levels in drug-naïve MDD patients [58, 59]. As a key brain region involved in emotional and cognitive regulation, the ovPFC is also a core area with pathological damage and functional abnormalities in MDD [60–62], suggesting that abnormal activation of the SAA/FPR2/CSF1/NLRP3 axis in this region may be an important molecular basis for central neuroinflammation in MDD. Additionally, studies have confirmed that peripheral SAA can cross the BBB to enter the CNS [57, 63, 64]. Our current and previous research [54, 65] has shown that LPS can induce increased expression of SAA1/2 in the brains of model animals, indicating that elevated peripheral SAA caused by inflammation may either enter the brain to activate central FPR2 or directly induce central SAA production, creating a crosstalk effect between peripheral and central inflammation. This also provides a previously unrecognized molecular pathway to explain how peripheral inflammatory signals are transmitted to the central nervous system in inflammatory depression.

This study still has several limitations. First, isolated brain microglia may be contaminated by peripherally infiltrating macrophages. Previous studies have reported peripheral macrophage infiltration in the brain in depressive pathology [6, 66]. The present study did not verify the specific contributions of these two cell populations to the SAA/FPR2/CSF1/NLRP3 axis-mediated depressive-like behaviors. Future research could address this by using bone marrow transplants and/or cell-specific knockout techniques. Given that FPR2 is predominantly expressed in microglia and markedly upregulated in these cells upon LPS challenge, the use of global *Fpr2*^–/–^ mice in this study provides a reasonable proof-of-concept at this stage, pending future validation with microglia-specific knockouts to definitively determine the contribution of microglial FPR2 versus other cell types. Second, the function of peripheral FPR2 in depression remains unaddressed. FPR2 is widely expressed in peripheral tissues and cells [24]. Subsequent studies can employ tissue-specific FPR2 knockout models to explore whether and how peripheral FPR2 contributes to depressive-like behaviors. Third, this study validated the role of this axis in the LPS-induced acute model of inflammatory depression. Since chronic stress is a major factor in the development of MDD in modern society [67], future research needs to verify the role and regulatory mechanisms of this axis in other classic depression models, such as chronic unpredictable stress and social defeat, to confirm its universality across different types of depression. Fourth, the RNA-seq samples used in this study were whole brains with the olfactory bulbs, cerebellum, and brainstem removed. Given that different brain regions play distinct roles in depression [68, 69], future single-cell or spatial transcriptomics studies could address region-specific contributions. Lastly, as a functionally complex receptor, FPR2 can mediate pro-inflammatory or anti-inflammatory effects depending on its ligand [24]. This study focused solely on the role of SAA. Future work can explore the expression of various endogenous ligands across different depressive models and determine whether and how imbalances in their expression contribute to the pathogenesis of depression via FPR2, thereby providing more comprehensive evidence for precisely targeting FPR2 modulation.

## Supplementary Information

Supplementary Material 1.

Supplementary Material 2.

Supplementary Material 3.

Supplementary Material 4.

## Data Availability

The datasets generated and analyzed in this study are available from the corresponding author upon reasonable request.

## Abbreviations

ACC: Anterior cingulate cortex
CA1: Cornu Ammonis 1
CA3: Cornu Ammonis 3
CNS: Central nervous system
CSF1: Colony-stimulating factor 1
DEGs: Differentially expressed genes
DG: Dentate gyrus
EPM: Elevated plus maze
FC: Fold change
FPR2: Formyl peptide receptor 2
FST: Forced swim test
GO: Gene ontology
GSEA: Gene set enrichment analysis
ICV: Intracerebroventricular
IL-1β: Interleukin-1 beta
LPS: Lipopolysaccharide
MDD: Major depressive disorder
NLRP3: Nod-like receptor protein 3
OFT: Open field test
PBS: Phosphate-buffered saline
PFC: Prefrontal cortex
SAA: Serum amyloid A
SPT: Sucrose preference test
SYN: Synaptophysin
TBS: Tris Buffered Saline
TST: Tail suspension test
WT: Wild-type
