# Supplementary material for: FPR2 deficiency alleviates LPS-induced depressive-like behaviors in mice by suppressing the microglial CSF1/NLRP3 inflammasome pathway
Source: J Neuroinflammation. 2026 Jul 16;23:338. doi: 10.1186/s12974-026-03963-4 (PMC13656527; doi:10.1186/s12974-026-03963-4)
Supplement: Supplementary file 1 [file 12974_2026_3963_MOESM4_ESM.pdf]

Figure 3K

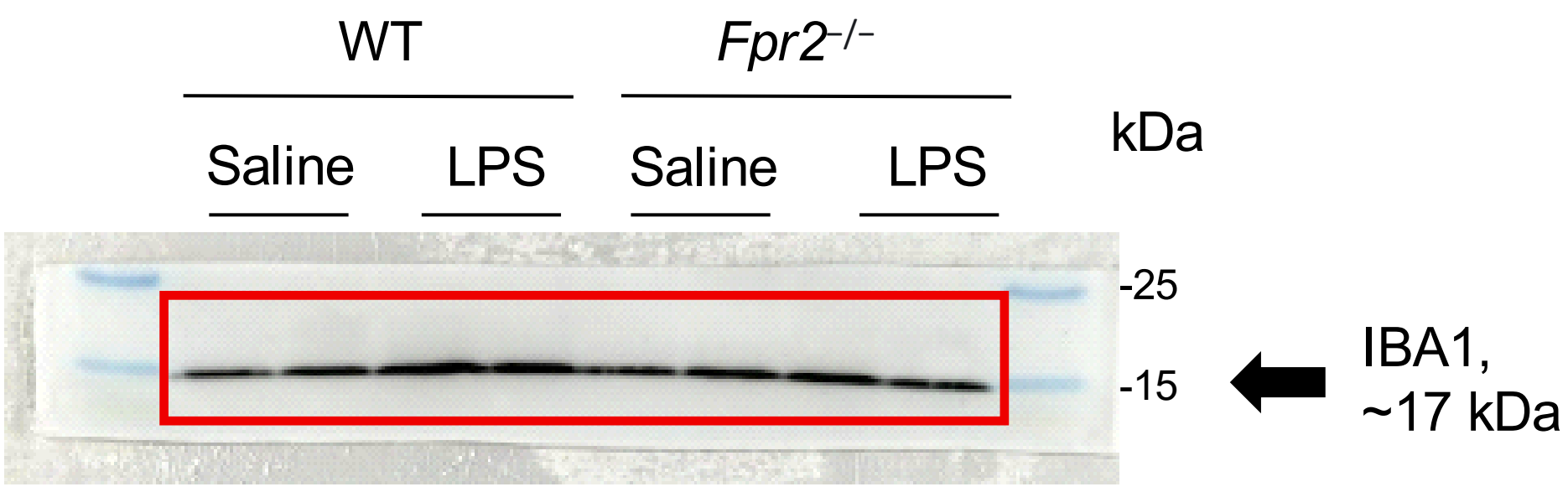
WT
Fpr2-/-
Saline
LPS
Saline
LPS
kDa
-25
-15
IBA1,
~17 kDa

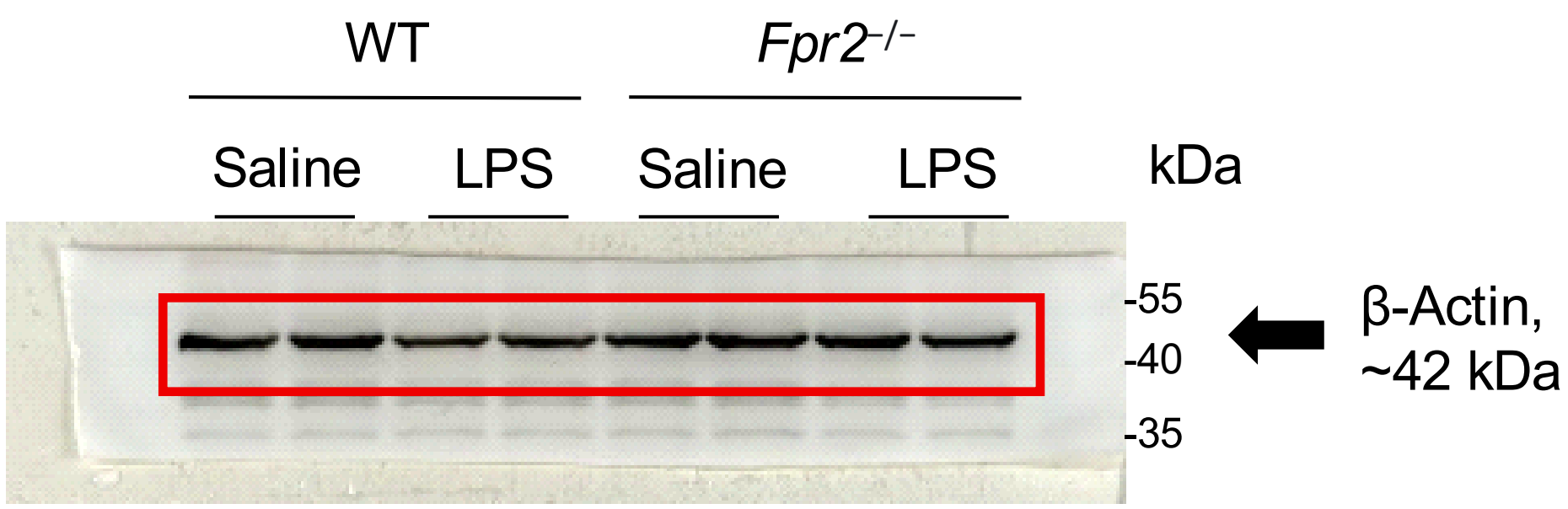
WT
Fpr2-/-
Saline
LPS
Saline
LPS
kDa
-55
-40
-35
β-Actin,
~42 kDa

Figure 3L

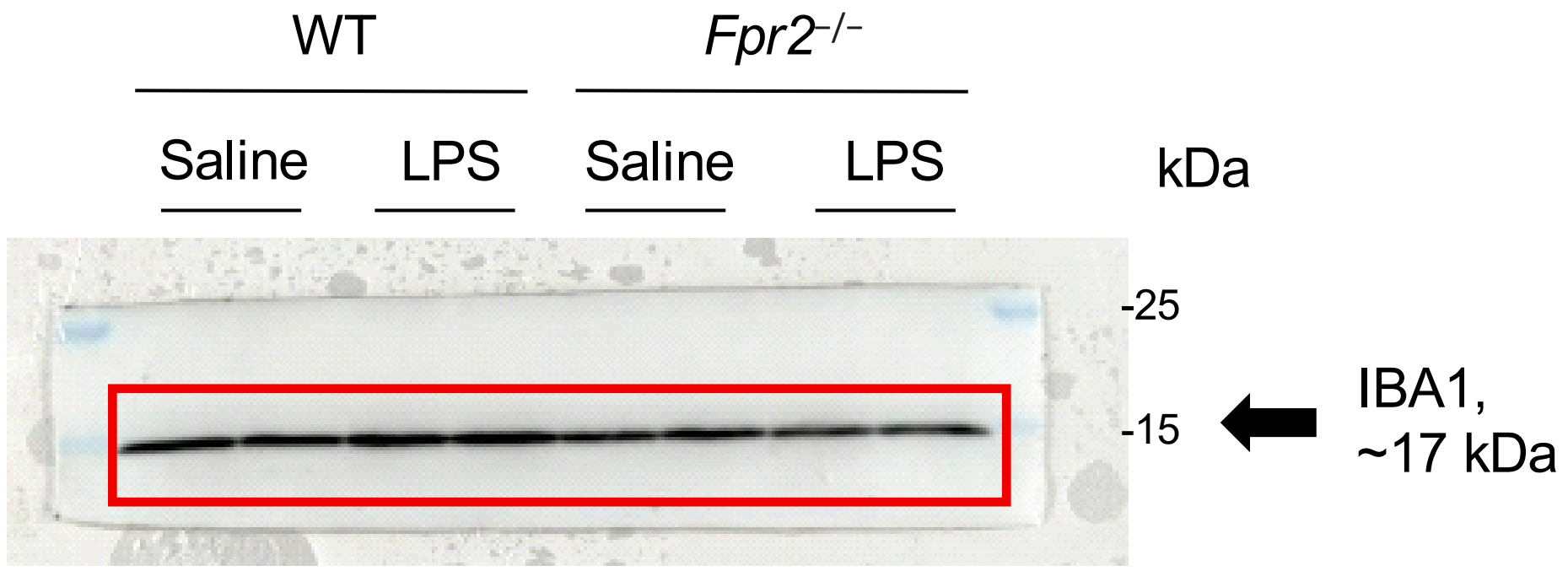
WT
Fpr2−/−
Saline
LPS
Saline
LPS
kDa
-25
-15
IBA1,
~17 kDa

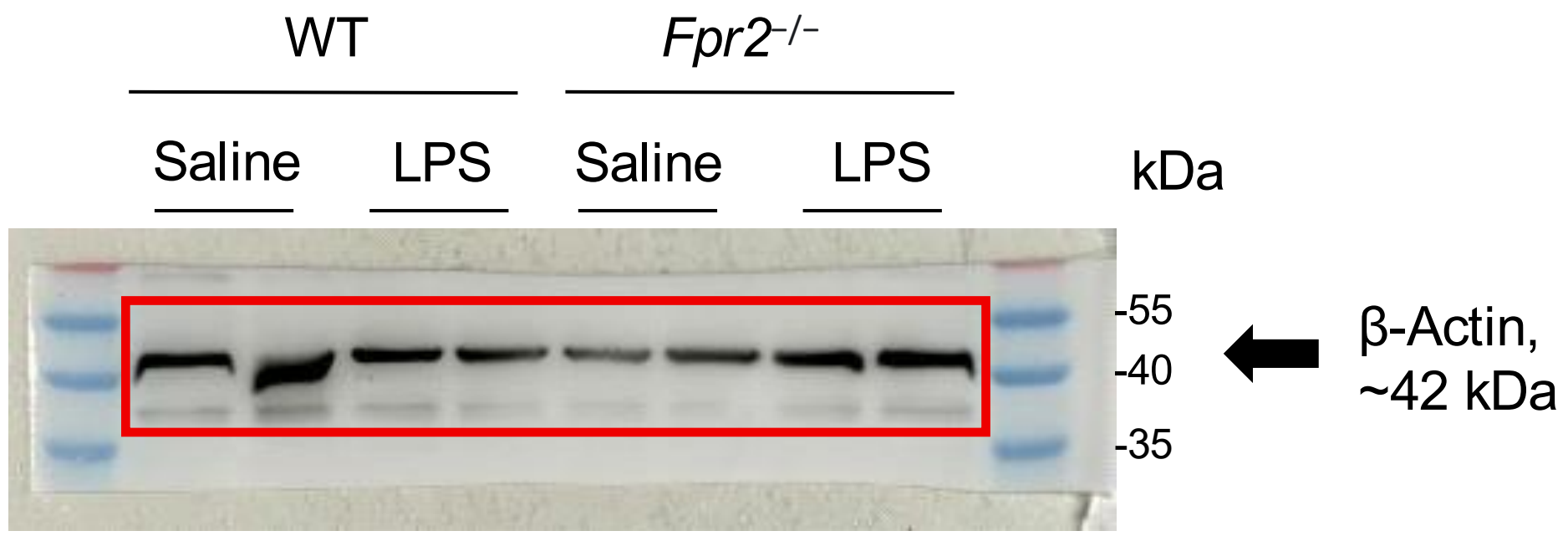
WT
Fpr2−/−
Saline
LPS
Saline
LPS
kDa
-55
-40
-35
β-Actin,
~42 kDa

Figure 7G

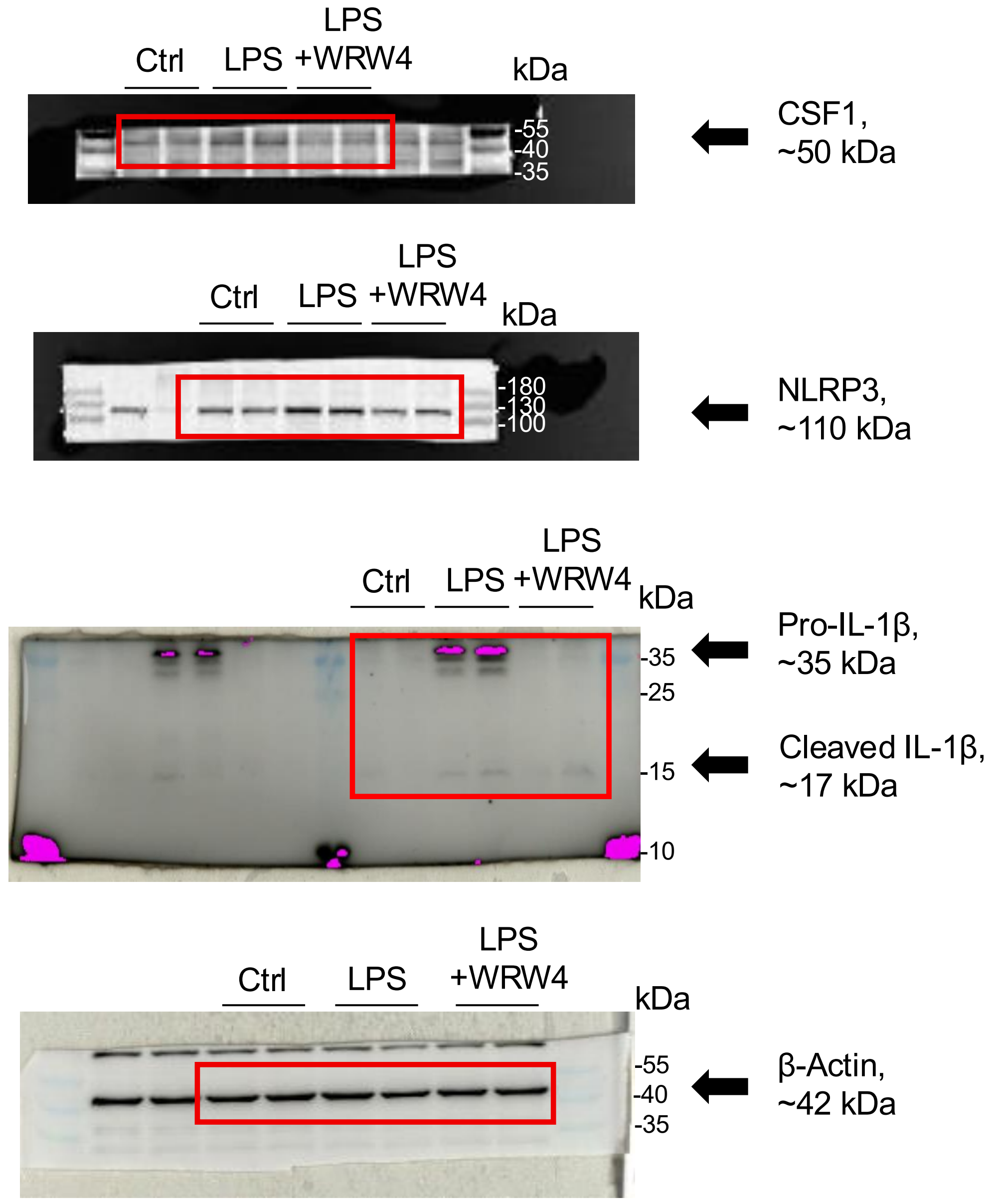
Ctrl
LPS
LPS +WRW4
kDa
-55
-40
-35
CSF1, ~50 kDa
Ctrl
LPS
LPS +WRW4
kDa
-180
-130
-100
NLRP3, ~110 kDa
Ctrl
LPS
LPS +WRW4
kDa
-35
-25
-15
-10
Pro-IL-1β, ~35 kDa
Cleaved IL-1β, ~17 kDa
Ctrl
LPS
LPS +WRW4
kDa
-55
-40
-35
β-Actin, ~42 kDa

Figure 7K

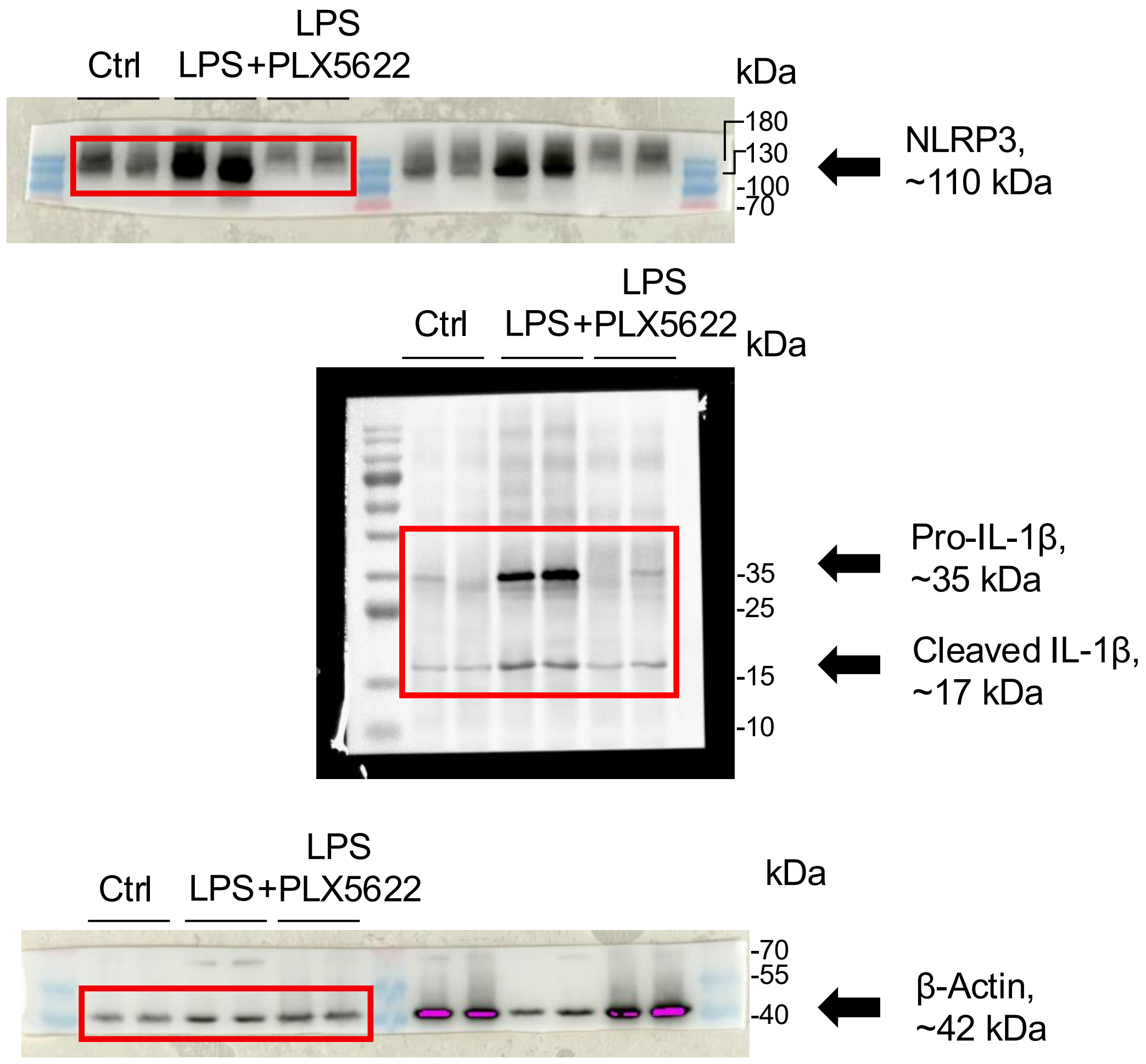
Ctrl
LPS
LPS+PLX5622
kDa
-180
-130
-100
-70
NLRP3,
~110 kDa
Ctrl
LPS
LPS+PLX5622
kDa
-35
-25
-15
-10
Pro-IL-1β,
~35 kDa
Cleaved IL-1β,
~17 kDa
Ctrl
LPS
LPS+PLX5622
kDa
-70
-55
-40
β-Actin,
~42 kDa

Figure 8A

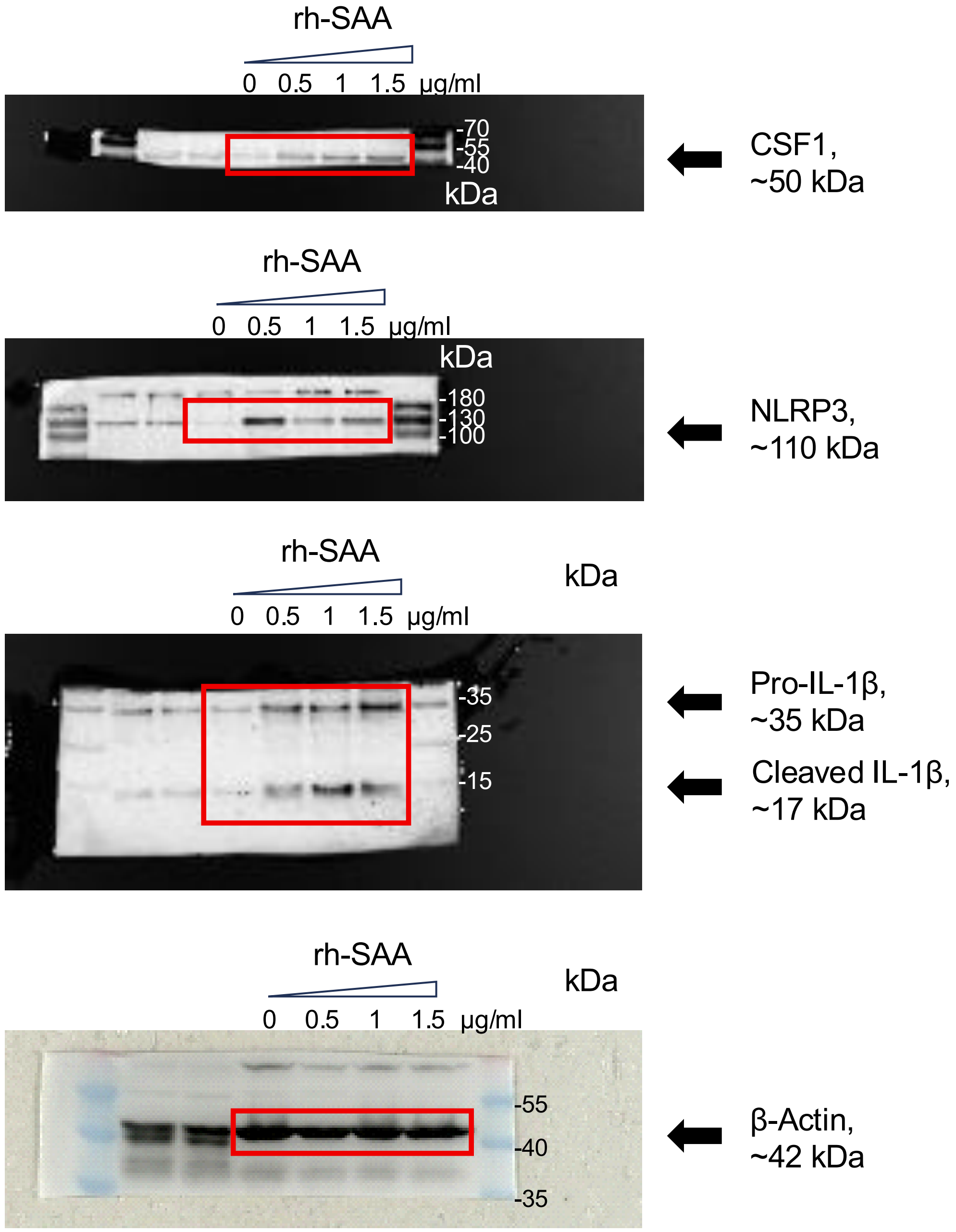
rh-SAA
0 0.5 1 1.5 µg/ml
-70
-55
-40
kDa
CSF1,
~50 kDa
rh-SAA
0 0.5 1 1.5 µg/ml
kDa
-180
-130
-100
NLRP3,
~110 kDa
rh-SAA
kDa
0 0.5 1 1.5 µg/ml
-35
-25
-15
Pro-IL-1β,
~35 kDa
Cleaved IL-1β,
~17 kDa
rh-SAA
kDa
0 0.5 1 1.5 µg/ml
-55
-40
-35
β-Actin,
~42 kDa

Figure 8C

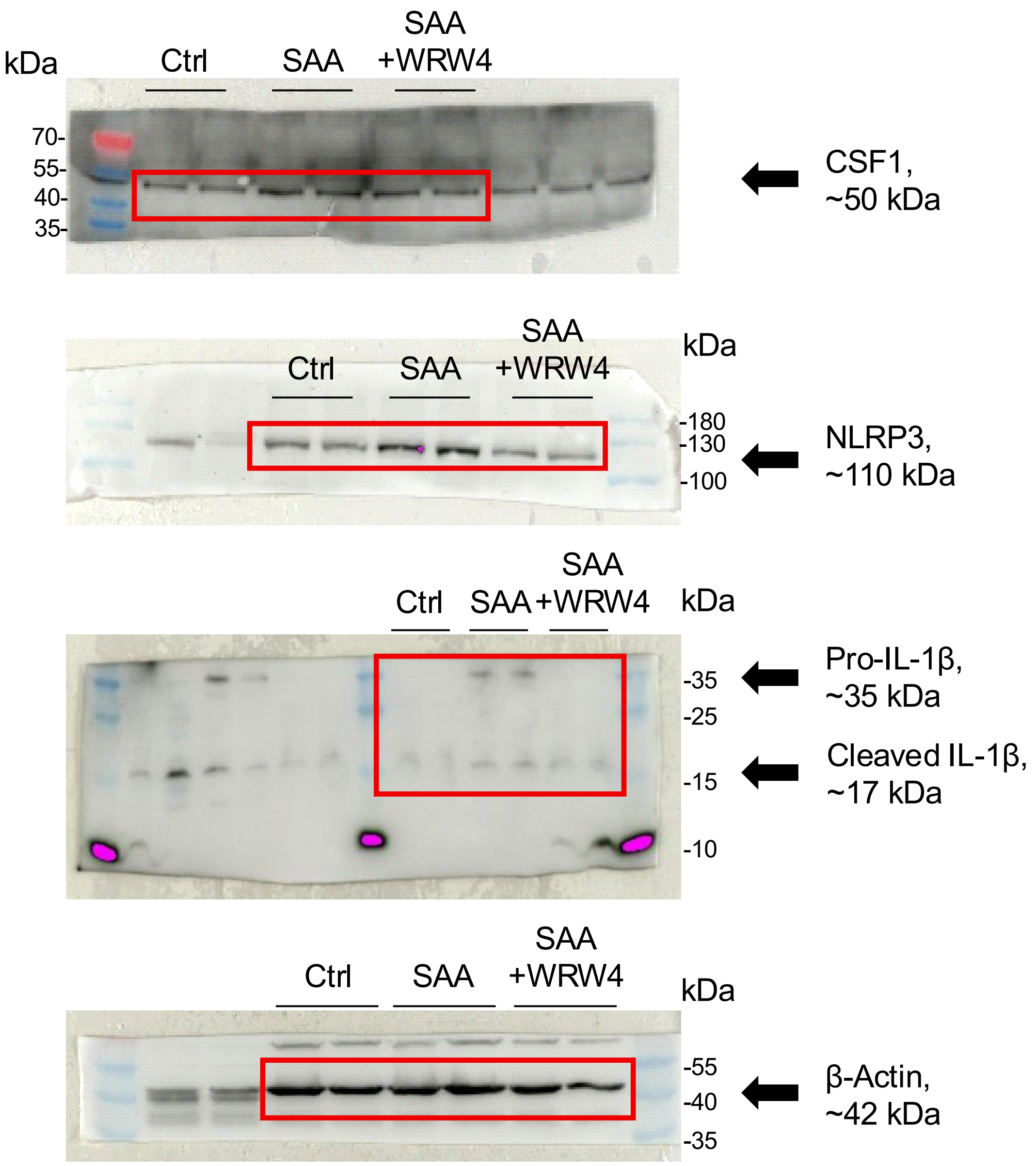

kDa
Ctrl
SAA
SAA +WRW4
70-
55-
40-
35-
CSF1, ~50 kDa
Ctrl
SAA
SAA +WRW4
kDa
-180
-130
-100
NLRP3, ~110 kDa
Ctrl
SAA
SAA +WRW4
kDa
-35
-25
-15
-10
Pro-IL-1β, ~35 kDa
Cleaved IL-1β, ~17 kDa
Ctrl
SAA
SAA +WRW4
kDa
-55
-40
-35
β-Actin, ~42 kDa

Figure 8G

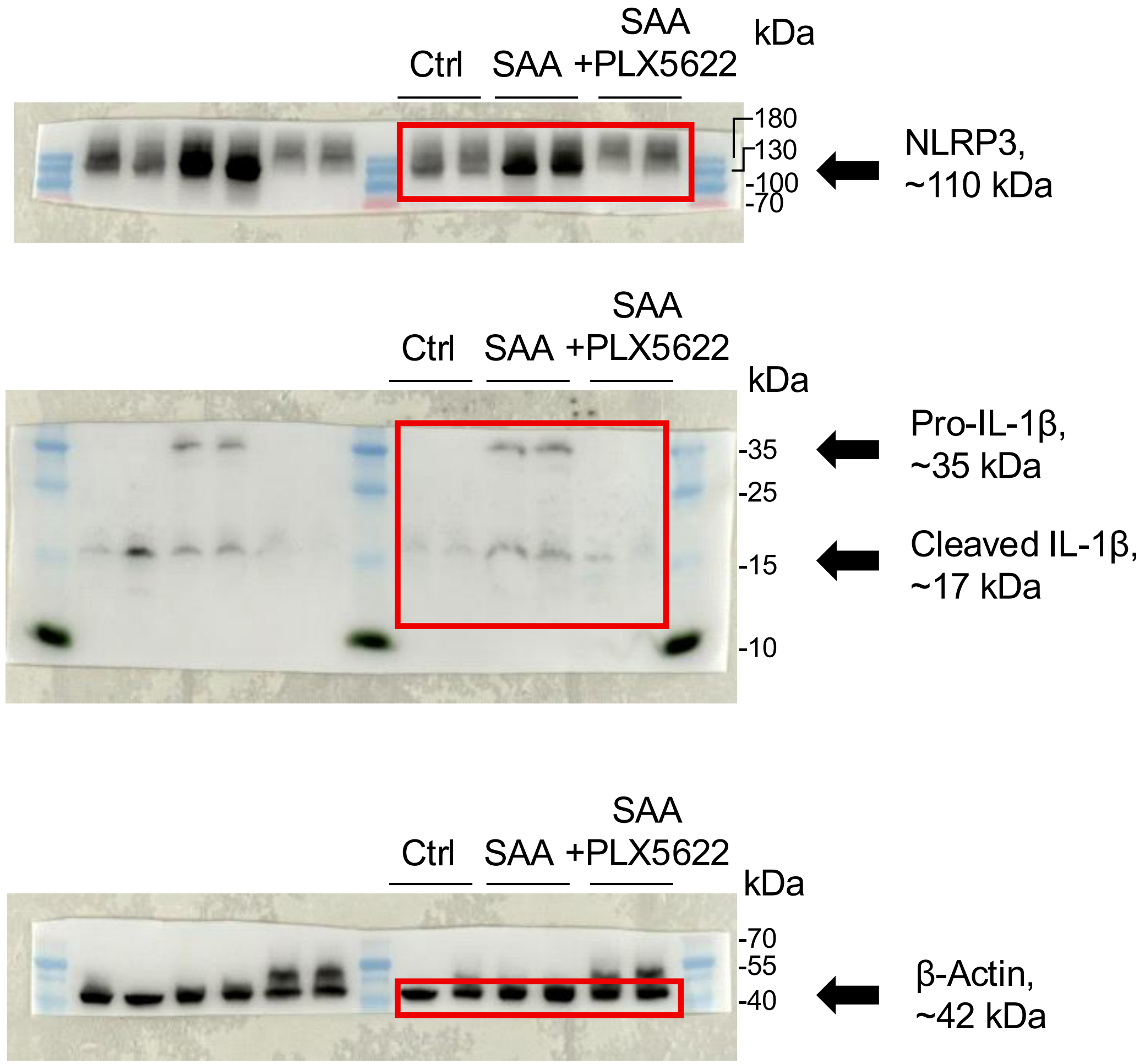
Ctrl
SAA
SAA +PLX5622
kDa
180
130
100
70
NLRP3, ~110 kDa
Ctrl
SAA
SAA +PLX5622
kDa
35
25
15
10
Pro-IL-1β, ~35 kDa
Cleaved IL-1β, ~17 kDa
Ctrl
SAA
SAA +PLX5622
kDa
70
55
40
β-Actin, ~42 kDa

Figure S9B

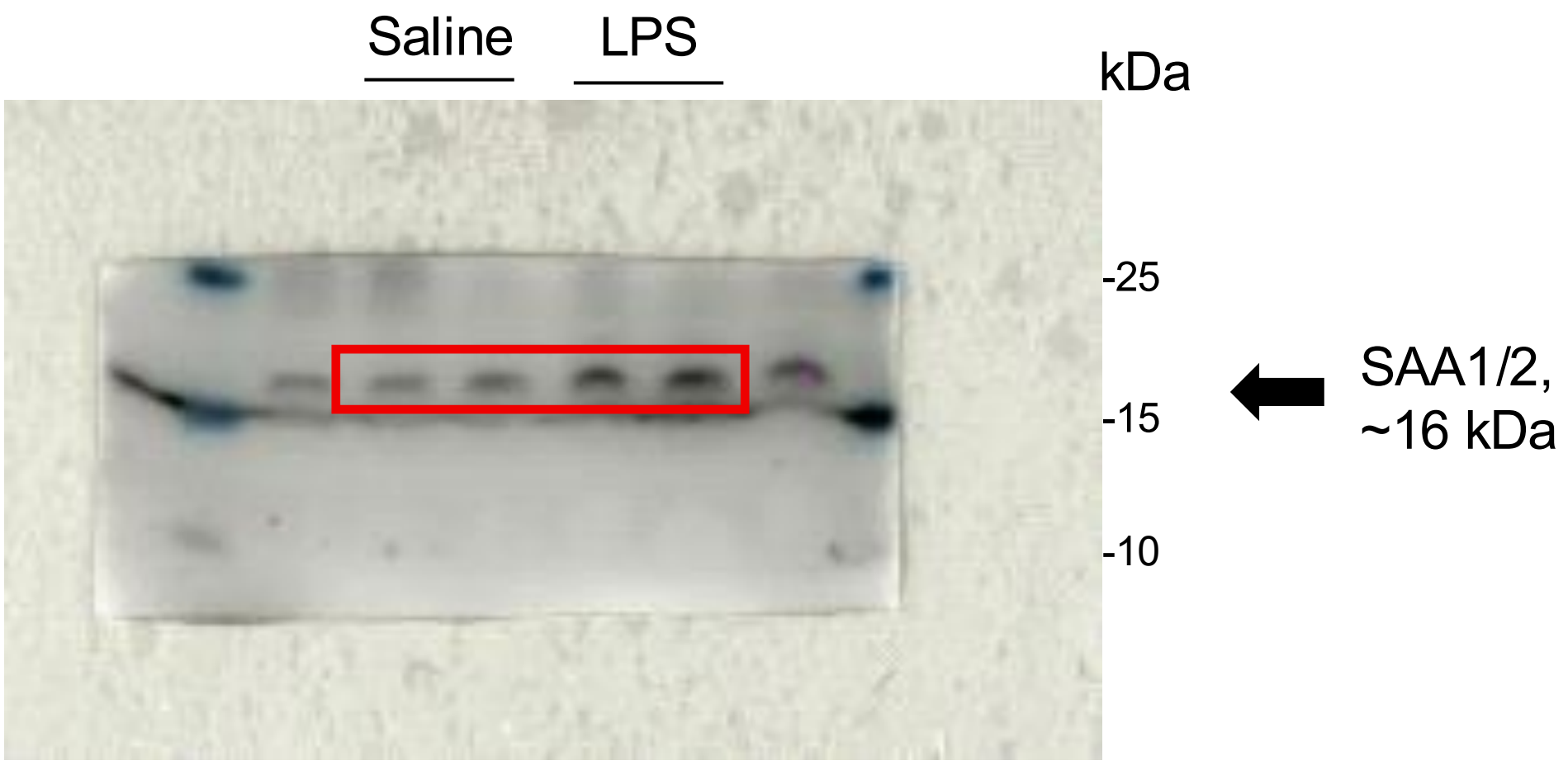

Saline
LPS
kDa
-25
-15
-10
SAA1/2,
~16 kDa

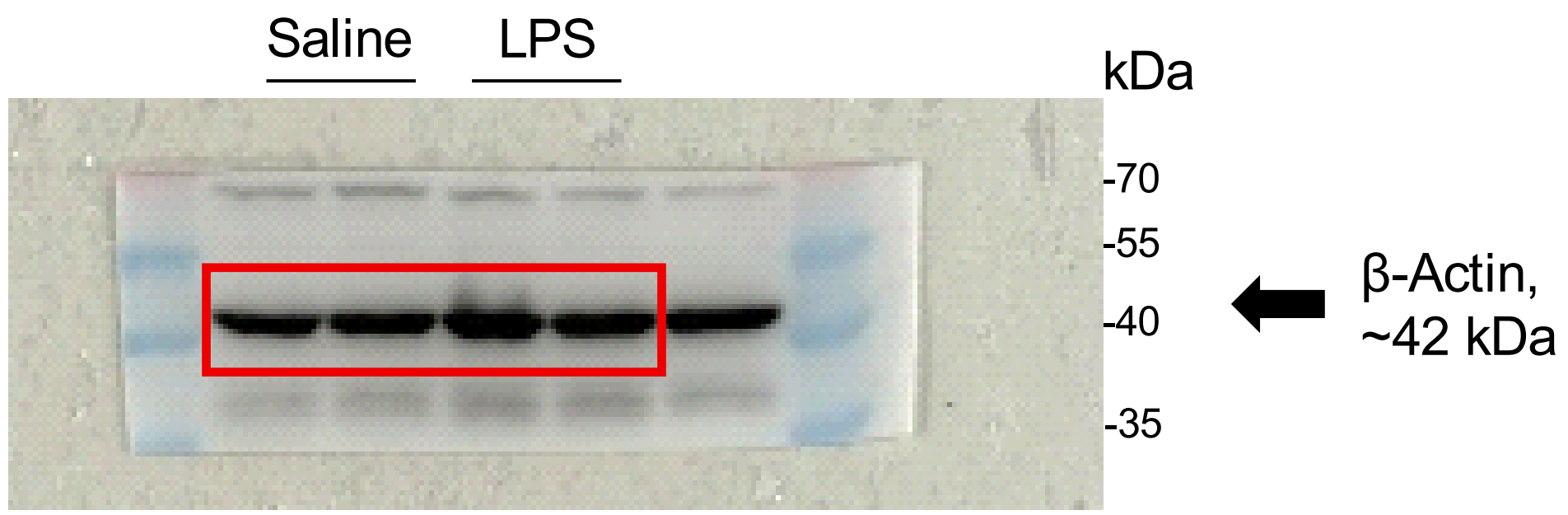

Saline
LPS
kDa
-70
-55
-40
-35
β-Actin,
~42 kDa
