# Supplementary material for: FPR2 deficiency alleviates LPS-induced depressive-like behaviors in mice by suppressing the microglial CSF1/NLRP3 inflammasome pathway
Source: J Neuroinflammation. 2026 Jul 16;23:338. doi: 10.1186/s12974-026-03963-4 (PMC13656527; doi:10.1186/s12974-026-03963-4)
Supplement: Supplementary file 2 [file 12974_2026_3963_MOESM1_ESM.docx]

**Additional file 1.**

**Supplementary Figures 1-12 and Tables 1-****5**

**Supplementary Figures**

**Figure S1.** FPR2 expression is significantly upregulated in microglia of the hippocampal CA1 and CA3 regions following LPS administration. **A, H.** Representative immunofluorescence images showing FPR2 expression in the hippocampal cornu ammonis-1 (CA1, **A**) and CA3 (**H**) regions of wild-type (WT) mice treated with LPS or saline. Scale bar, 50 μm. **B–E, I–L.** Quantification of FPR2 fluorescence intensity (**B, I**), Manders’ coefficient M1 (the fraction of IBA1 colocalized with FPR2, **C, J**), the percentage of FPR2^+^IBA1^+^ cells among IBA1⁺ cells (**D, K**), and the area of FPR2 colocalized with IBA1 (**E, L**) in the hippocampal CA1 (**B–E**) and CA3 (**I–L**). **F–G, M–N.** Line scan analysis of FPR2 and IBA1 fluorescence intensity along a 30 μm segment in the hippocampal CA1 (**F, G**) and CA3 (**M, N**) of mice treated with saline (WT-Saline) or LPS (WT-LPS). Data are presented as means ± SEM. For each region, 1-2 individual fields were analyzed per mouse, with *n* = 3 mice per group. Statistical comparisons between groups are indicated by lines with *p*‑values above.

**Figure S2.** FPR2 expression is upregulated more significantly in microglia than in neurons after LPS treatment. **A, D.** Representative immunofluorescence images showing FPR2 expression in microglia (IBA1^+^) and neurons (NeuN^+^) in the prefrontal cortex (PFC, **A**) and hippocampus (**D**) of WT-Saline and WT-LPS mice. White arrows indicate neurons, and yellow arrows indicate microglia. Scale bars: 50 μm (**A**), 100 μm (**D**). **B, E.** Percentage of FPR2^+^NeuN^+^ cells in the PFC (**B**) and hippocampus (**E**). Quantification of FPR2 fluorescence intensity in IBA1^+^ and NeuN^+^ cells in the PFC (**C**) and hippocampus (**F**). Data are presented as means ± SEM, based on two individual fields for each region (**B, E**) or 12 cells for each region (**C, F**), with *n* = 3 mice per group. A two-tailed unpaired Student’s t-test was used to compare two groups (**B, E**). Two-way ANOVA followed by Tukey’s post hoc test was used for multiple comparisons (**C, F**). Statistical comparisons between groups are indicated by lines with *p*‑values above.

**Figure S3.** FPR2 deficiency mitigates LPS-induced microglial activation in the hippocampal CA1 and CA3 regions. **A, F.** Representative immunofluorescence and skeletonization images of microglia in the hippocampal cornu ammonis-1 (CA1, **A**) and CA3 (**F**) regions of wild-type (WT) and *Fpr2*^–/–^ mice 24 h after LPS or saline administration. Scale bar: 20 μm. **B–E, G–J.** Quantification of IBA1 fluorescence intensity (**B, G**), number of IBA1⁺ cells (**C, H**), microglial soma area (**D, I**), and Sholl analysis of process intersections (**E, J**) in the hippocampal CA1 (**B–E**) and CA3 (**G–J**) regions. Data are presented as means ± SEM. For each region: 2 individual fields were analyzed per mouse for IBA1 intensity and cell count (**B–C, G–H**); 10-14 cells for soma area (**D, I**); 8 cells for Sholl analysis (**E, J**); *n* = 3 mice per group. One-way ANOVA (B–D, G–I) and two-way ANOVA (E, J), followed by Tukey’s post hoc test, were used for multiple comparisons. Statistical comparisons between groups are indicated by lines with *p*‑values above. * *p* < 0.05, **** *p* < 0.0001 for WT-LPS vs. WT-saline group. ^#^ *p* < 0.05, ^####^ *p* < 0.0001 for *Fpr2*^–/–^-LPS vs. WT-LPS group.

**Figure S4.** Microglial activation positively correlates with FPR2 expression in the PFC and hippocampus. Correlation between IBA1 and FPR2 fluorescence intensity in the prefrontal cortex (PFC) and in the hippocampal dentate gyrus (DG), cornu ammonis-1 (CA1), and CA3 regions of WT-Saline and WT-LPS mice. Data are presented as individual data points. Correlation coefficients (R) and *p*-values were determined by two-tailed Spearman correlation analysis. *n* = 3 mice per group.

**Figure S5.** FPR2 deficiency suppresses activation of the NLRP3 inflammasome signaling pathway in the hippocampal CA1 and CA3 regions. **A, F.** Representative immunofluorescence images showing NLRP3 expression in the hippocampal cornu ammonis-1 (CA1, **A**) and CA3 (**F**) regions of WT and *Fpr2*^–/–^ mice after LPS or saline administration. Scale bar: 40 μm. **B–E, G–J.** Quantification of NLRP3 fluorescence intensity (**B, G**), the area of NLRP3 colocalized with IBA1 (**C, H**), the percentage of NLRP3⁺IBA1⁺ cells among IBA1⁺ cells (**D, I**), and Manders’ coefficient M2 (the fraction of NLRP3 colocalized with IBA1, **E, J**) in the hippocampal CA1 (**B–E**) and CA3 (**G–J**) regions. **K, P.** Representative immunofluorescence images showing IL-1β expression in the hippocampal CA1 (**K**) and CA3 (**P**) of WT and *Fpr2*^–/–^ mice after LPS or saline administration. **L–O, Q–T.** Quantification of IL-1β fluorescence intensity (**L, Q**), the area of IL-1β colocalized with IBA1 (**M, R**), the percentage of IL-1β⁺IBA1⁺ cells among IBA1⁺ cells (**N, S**), and Manders’ coefficient M2 (the fraction of IL-1β colocalized with IBA1, **O, T**) in the hippocampal CA1 (**L–O**) and CA3 (**Q–T**). Data are presented as means ± SEM. For each region, 1–2 individual fields were analyzed per mouse, with *n* = 3 mice per group. One-way ANOVA followed by Tukey’s post hoc test was used for multiple comparisons. Statistical comparisons between groups are indicated by lines with *p*‑values above.

**Figure S6.** FPR2 deficiency reduces LPS-induced *Il1b* upregulation in microglia. RT-qPCR validation of *Il1b* transcript levels in microglia from WT-Saline, WT-LPS, and *Fpr2*^–/–^-LPS mice. Data are presented as mean ± SEM, *n* = 3 mice per group. One-way ANOVA followed by Tukey’s post hoc test was used for multiple comparisons. Statistical comparisons between groups are indicated by lines with *p*‑values above.

**Figure S7.** FPR2 deficiency prevents LPS-induced CSF1 upregulation in microglia within the hippocampal CA1 and CA3 regions. **A–C.** Representative immunofluorescence images (**A**) and quantification (**B, C**) of CSF1 expression and colocalization with IBA1 in the hippocampal cornu ammonis-1 (CA1) of WT and *Fpr2*^–/–^ mice after LPS or saline administration. Scale bar, 10 μm. **D–F.** Representative immunofluorescence images (**D**) and quantification (**E, F**) of CSF1 expression and colocalization with IBA1 in the hippocampal CA3 of WT and *Fpr2*^–/–^ mice after LPS or saline administration. Scale bar, 10 μm. Data are presented as means ± SEM. Two individual fields were analyzed per mouse for each region, with *n* = 3 mice per group. One-way ANOVA followed by Tukey’s post hoc test was used for multiple comparisons. Statistical comparisons between groups are indicated by lines with *p*‑values above.

**Figure S8.** SAA activates the NLRP3 inflammasome pathway in microglia. Representative immunofluorescence images (left) and quantification (right) of CSF1, NLRP3, and IL-1β levels in N9 cells treated with media alone (control, Ctrl), 1.5 μg/ml SAA, or 2.5 ng/ml LPS. Scale bar, 10 μm. Data are presented as means ± SEM. *n* = 3 independent cell cultures per group. One-way ANOVA followed by Tukey’s post hoc test was used for multiple comparisons. Statistical comparisons between groups are indicated by lines with *p*‑values above. SAA: recombinant human serum amyloid A 1/2 (rh-SAA).

**Figure S9.** SAA activates NLRP3 in microglia via FPR2 and CSF1R in the hippocampal CA1 and CA3 regions. **A, F.** Representative immunofluorescence images showing NLRP3 expression in the hippocampal cornu ammonis-1 (CA1, **A**) and CA3 (**F**) regions of WT and *Fpr2*^–/–^ mice after ICV injection of saline, rh‑SAA, or rh‑SAA with PLX5622. Scale bar: 10 μm. **B–E**, **G–J**. Quantification of NLRP3 fluorescence intensity (**B, G**), the area of NLRP3 colocalized with IBA1 (**C, H**), the percentage of NLRP3⁺IBA1⁺ cells among IBA1⁺ cells (**D, I**), and Manders’ coefficient M2 (the fraction of NLRP3 colocalized with IBA1, **E, J**) in the hippocampal CA1 (**B–E**) and CA3 (**G–J**) regions. Data are presented as means ± SEM. For each region, 1–2 individual fields were analyzed per mouse, with *n* = 3 mice per group. One‑way ANOVA followed by Tukey’s post hoc test was used for multiple comparisons. Statistical comparisons between groups are indicated by lines with *p*‑values above. SAA: recombinant human serum amyloid A 1/2 (rh-SAA).

**Figure S10.** LPS induces SAA1/2 expression in the mouse brain. **A.** RT-qPCR analysis of *Saa1* and *Saa2* transcript levels in the brains of WT-Saline and WT-LPS mice. **B.** Western blot analysis (left) and quantification (right) of SAA1/2 protein expression in the brains of WT-Saline and WT-LPS mice. Data are presented as mean ± SEM. *n* = 3 mice per group. A two-tailed unpaired Student’s t-test was used to compare the two groups. Statistical comparisons between groups are indicated by lines with *p*‑values above.

**Figure S11.** SAA1 protein levels in the plasma of patients with postpartum depression and vascular depression. Proteomic analysis of SAA1 abundance in plasma from postpartum depression (PPD) patients versus healthy controls (dataset PXD050851) and from vascular depression (VaD) patients versus healthy controls (dataset PXD047302). Statistical comparisons between groups are indicated by lines with *p*‑values above.

**Figure S12.** Top 10 enriched biological process (BP) terms from the GO analysis of dataset PXD034030. Asterisks indicate terms associated with immune or inflammatory responses (eight of the top ten terms). Enriched terms involving SAA1 include regulation of response to external stimulus, acute inflammatory response, response to stress, and wound healing.

**Supplementary** **Tables**

**Table S1** Immunofluorescence staining protocols

| Staining | Antibody incubations (sequential order from left to right) |
| --- | --- |
| Single labeling |  |
| IBA1 | Iba1 (1:500, rabbit) → Alex Fluor (AF)647 anti-rabbit (1:500) |
| CSF1 | CSF1 (1:100, rabbit) → AF647 anti-rabbit (1:500) |
| NLRP3 | NLRP3-FITC (1:200, directly conjugated) |
| IL-1β | IL-1β-FITC (1:200, directly conjugated) |
| Double labeling |  |
| FPR2/IBA1 | Iba1 (1:500, rabbit) → AF568 anti-rabbit (1:500) → FPR2-AF647 (1:200) |
| SYN/NeuN | NeuN (1:500, mouse) → AF568 anti-mouse (1:500) → Synaptophysin (1:500, rabbit) → AF647 anti-rabbit (1:500) |
| NLRP3/IBA1 | Iba1 (1:500, rabbit) → AF647 anti-rabbit (1:500) → NLRP3-FITC (1:200) |
| IL-1β/IBA1 | Iba1 (1:500, rabbit) → AF647 anti-rabbit (1:500) → IL-1β-FITC (1:200) |
| CSF1/IBA1 | CSF1 (1:100, rabbit) → AF647 anti-rabbit (1:500) → Iba1-AF488 (1:100) |
| Triple labeling |  |
| FPR2/NeuN/IBA1 | NeuN (1:500, mouse) → AF488 anti-mouse (1:500) → Iba1 (1:500, rabbit) → AF568 anti-rabbit (1:500) → FPR2-AF647 (1:200) |

**Table S2** Top 10 hub genes identified from FPR2 deficiency-reversed DEGs in LPS-stimulated mouse brain microglia using the Radiality method in cytoHubba

| **Rank** | **Name** | **Score** |
| --- | --- | --- |
| 1 | *Csf1* | 0.9191919191919191 |
| 1 | *Traf6* | 0.9191919191919191 |
| 3 | *Nlrp3* | 0.8484848484848484 |
| 4 | *Itgb3* | 0.813131313131313 |
| 5 | *Birc3* | 0.7777777777777777 |
| 5 | *Tnip1* | 0.7777777777777777 |
| 7 | *Xbp1* | 0.6545454545454545 |
| 7 | *Pdia4* | 0.6545454545454545 |
| 9 | *Anpep* | 0.6363636363636362 |
| 10 | *Creld2* | 0.6181818181818182 |

Note: Protein-protein interaction (PPI) networks for the 70 FPR2 deficiency-reversed differentially expressed genes (DEGs) were constructed using the STRING database (confidence score > 0.4) and visualized with Cytoscape (version 3.10.2). Hub genes were identified by topological analysis using the cytoHubba plugin [1]; the top 10 genes ranked by the Radiality method are listed with their corresponding scores.

**Table S3** Topological features of hub genes identified from FPR2 deficiency-reversed DEGs in LPS-stimulated mouse brain microglia using the closeness method in CytoNCA

| **Rank** | **Name** | **Score** |
| --- | --- | --- |
| 1 | *Csf1* | 0.03690888 |
| 2 | *Traf6* | 0.03690888 |
| 3 | *Nlrp3* | 0.036823936 |
| 4 | *Itgb3* | 0.03678161 |
| 5 | *Tnip1* | 0.03673938 |
| 6 | *Birc3* | 0.03673938 |
| 7 | *Anpep* | 0.03657143 |
| 8 | *Pdia4* | 0.03567447 |
| 9 | *Xbp1* | 0.03567447 |
| 10 | *Hyou1* | 0.035634745 |
| 11 | *Creld2* | 0.035634745 |
| 12 | *Ppp1r15b* | 0.035516094 |
| 13 | *Tubb6* | 0.035516094 |
| 14 | *Eif2s3y* | 0.03448276 |
| 15 | *Uty* | 0.03444564 |
| 16 | *Kdm5d* | 0.03444564 |
| 17 | *Ddx3y* | 0.03444564 |
| 18 | *Etf1* | 0.034371644 |
| 19 | *Mmp8* | 0.033333335 |
| 20 | *Eln* | 0.03329865 |
| 21 | *Elane* | 0.03329865 |
| 22 | *Il1r2* | 0.033264033 |
| 23 | *C9orf72* | 0.032258064 |
| 24 | *Rasgrp3* | 0.03222558 |
| 25 | *Sesn2* | 0.03222558 |
| 26 | *Rabgef1* | 0.03125 |
| 27 | *Rab20* | 0.03125 |
| 28 | *Rcan1* | 0.03125 |
| 29 | *Ets2* | 0.03125 |
| 30 | *Cpeb4* | 0.03125 |
| 31 | *Cpeb2* | 0.03125 |
| 32 | *Gas2l3* | 0.03125 |
| 33 | *Ckap2l* | 0.03125 |

Note: Protein-protein interaction (PPI) networks for the 70 FPR2 deficiency-reversed differentially expressed genes (DEGs) were constructed using the STRING database (confidence score > 0.4) and visualized with Cytoscape. Hub genes were identified by topological analysis using the CytoNCA plugin [2].

**Table S4** Primers used for RT-qPCR

| Gene | Forward primer (5' to 3') | Reverse primer (5' to 3') |
| --- | --- | --- |
| *Nlrp3* | AGGAGGAAGAAGAAGAGAGGAGAGG | TTGAGAAGAGACCACGGCAGAAG |
| *Il1b* | AATCTCGCAGCAGCACATCAAC | AGGTCCACGGGAAAGACACAG |
| *Csf1* | CAATGCTAACGCCACCGAGAG | CTTGTTCTGCTCCTCATAGTCCTTG |
| *Saa1* | GGACACCATTGCTGACCAGGAAG | CAGGCAGTCCAGGAGGTCTGTAG |
| *Saa2* | GGGGAGTCTGGGCTGCTGAG | TGGTGTCCTCGTGTCCTCTGC |
| *Actb* (β-actin) | GGCTGTATTCCCCTCCATCG | CCAGTTGGTAACAATGCCATGT |

**Table S5** Characteristics of the included public proteomic databases from depressed individuals

| **Accession number** | **Type of depression** | **Sample** | **Number (patients, controls)** | **Gender (% Male; patients, controls)** | **Age^d^ (patients, controls)** | **Diagnosis (Scales)** | **Treatments** | **Authors (publication year)** |
| --- | --- | --- | --- | --- | --- | --- | --- | --- |
| PXD034030 | LLD^a^ | Plasma | 19, 31 | 15.8, 9.7 | 74.0 ± 6.9,  70.3 ± 6.4 | HAMD-21^e^ = 18.7 ± 5.2  GAD-7^f^ = 8.7 ± 6.3 | No | Silva-Costa et al. (2022) [3] |
| PXD047302 | VaD^b^ | Serum | 35, 36 | 57.1, 72.2 | 64.1 ± 7.1,  62.1 ± 5.1 | HAMD-17^g^ = 15.6 ± 5.3 | No^i^ | Lan et al. (2024) [4] |
| PXD050851 | PPD^c^ | Plasma | 30, 30 | 0.0, 0.0 | 31.0 ± 4.1,  31.0 ± 2.0 | EPDS^h^ ≥ 13 | No | Wang et al. (2025) [5] |

^a^LLD: Late-life depression; ^b^VaD: Vascular depression; ^c^PPD: Postpartum depression; ^d^Data are presented as mean ± SD; ^e^HAMD-21: 21-item Hamilton Depression Rating Scale; ^f^GAD-7: 7-item Generalized Anxiety Disorder score; ^g^HAMD-17: 17-item Hamilton Depression Rating Scale; ^h^EPDS: Edinburgh Postnatal Depression Scale; ^i^Free from antidepressants or antipsychotics use for at least 3 months prior to enrollment.

**References**

1.	Chin CH, Chen SH, Wu HH, Ho CW, Ko MT, Lin CY: cytoHubba: identifying hub objects and sub-networks from complex interactome. *BMC Syst Biol* 2014, 8 Suppl 4:S11.

2.	Tang Y, Li M, Wang J, Pan Y, Wu FX: CytoNCA: a cytoscape plugin for centrality analysis and evaluation of protein interaction networks. *Biosystems* 2015, 127:67-72.

3.	Silva-Costa LC, Smith BJ, Carregari VC, Souza G, Vieira EM, Mendes-Silva AP, de Almeida V, Carvalho BS, Diniz BS, Martins-de-Souza D: Plasma proteomic signature of major depressive episode in the elderly. *J Proteomics* 2022, 269:104713.

4.	Lan L, Peng S, Zhang R, He H, Yang Y, Xi B, Zhang J: Serum proteomic biomarker investigation of vascular depression using data-independent acquisition: a pilot study. *Front Aging Neurosci* 2024, 16:1341374.

5.	Wang S, Xu R, Li G, Liu S, Zhu J, Gao P: A Plasma Proteomics-Based Model for Identifying the Risk of Postpartum Depression Using Machine Learning. *J Proteome Res* 2025, 24:824-833.
